# An Efficient Kinetic Model for Assemblies of Amyloid Fibrils and Its Application to Polyglutamine Aggregation

**DOI:** 10.1371/journal.pone.0043273

**Published:** 2012-11-13

**Authors:** Stéphanie Prigent, Annabelle Ballesta, Frédérique Charles, Natacha Lenuzza, Pierre Gabriel, Léon Matar Tine, Human Rezaei, Marie Doumic

**Affiliations:** 1 Institut National de Recherche Agronomique, Jouy-en-Josas, France; 2 Institut National de Recherche en Informatique et Automatique, Rocquencourt, France; 3 Université Pierre et Marie Curie, Paris, France; 4 Commissariat à l’Energie Atomique, Gif-sur-Yvette, France; 5 Institut National de Recherche en Informatique et Automatique Rhônes-Alpes, Lyon, France; 6 Université Gaston Berger, Saint-Louis, Sénégal; National Center of Neurology and Psychiatry, Japan

## Abstract

Protein polymerization consists in the aggregation of single monomers into polymers that may fragment. Fibrils assembly is a key process in amyloid diseases. Up to now, protein aggregation was commonly mathematically simulated by a polymer size-structured ordinary differential equations (ODE) system, which is infinite by definition and therefore leads to high computational costs. Moreover, this Ordinary Differential Equation-based modeling approach implies biological assumptions that may be difficult to justify in the general case. For example, whereas several ordinary differential equation models use the assumption that polymerization would occur at a constant rate independently of polymer size, it cannot be applied to certain protein aggregation mechanisms. Here, we propose a novel and efficient analytical method, capable of modelling and simulating amyloid aggregation processes. This alternative approach consists of an integro-Partial Differential Equation (PDE) model of coalescence-fragmentation type that was mathematically derived from the infinite differential system by asymptotic analysis. To illustrate the efficiency of our approach, we applied it to aggregation experiments on polyglutamine polymers that are involved in Huntington’s disease. Our model demonstrates the existence of a monomeric structural intermediate 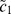 acting as a nucleus and deriving from a non polymerizing monomer (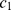). Furthermore, we compared our model to previously published works carried out in different contexts and proved its accuracy to describe other amyloid aggregation processes.

## Introduction

Protein aggregation and misfolding are involved in several fatal human disorders, such as Alzheimer’s, Prion, Huntington’s diseases [1], [2]. Certain types of aggregates display specific structural traits (*e.g.* a 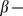
sheet enriched secondary structure) that characterize amyloid assemblies. Recent progress in solid state Nuclear Magnetic Resonance (NMR) has led to a better understanding of amyloid assemblies at the molecular level [3]. However, this structural knowledge constitutes only a snapshot of the dynamic processes. Protein aggregation involves a large amount of chain reactions, *e.g.* conformational exchange, nucleation (which is the formation of a first stable intermediate), polymerization by monomer, dimer or 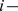 mer addition, coalescence, depolymerization (by loss of mono, di or oligomers), fragmentation (breakage into two or more polymers), protein degradation.

To explore the dynamics of amyloid assemblies, nucleation/polymerization reaction schemes have been applied, and to model them, ordinary differential equations (ODEs) have been used for many years [4]. An ODE means an equation containing only one independent variable (e.g. the chemical concentration of molecules) and its derivatives. Therefore in the case of polymerization, the number of equations should be at least equal to the number of sub-units constituting the longuest polymer. This value is extremely large in the case of amyloid fibrils (amyloid fibril sizes can go up to 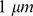 length [5]), therefore simplifying assumptions are commonly admitted, e.g. constant reaction rates, meaning that polymers of any size behave roughly in the same way [6]–[9]. Although such assumptions allow the system to be reduced from an infinite set of ODEs to a couple of equations [4], [7], assumptions of this nature are difficult to justify biochemically.

We propose here a new and global framework that can be adapted to most protein polymerization reactions. This method relies on partial differential equations (PDEs). In contrast to an ODE, a PDE permits formulation of problems involving functions of several variables. Instead of handling an infinite set of ODEs, we show that under reasonable assumptions, we can derive an equivalent model composed of a small number of ODEs coupled with a single size-structured PDE. The size variable of fibers replaces the infinite number of ODEs. To derive our model, we tune asymptotic methods from previously published works [10], [11]. A fully general model, which is much easier to handle both theoretically and numerically, is obtained. It allows much faster computations than for the full ODE set of equations. Moreover, recent analytical tools developed for PDE analysis can be applied. The obtention of size-distributions of polymers is a fundamental step [12], as it makes it possible to estimate quantitative reaction rates and build a predictive model by the means of recently developed inverse problem techniques [13].

To illustrate our method, we first formally derive the PDE model in a general case, and then apply our method to expanded polyglutamine (PolyQ) diseases. Finally, we compare our results to existing work [7], [8].

## Results

### The Infinite ODE System

Let us first recall how one can write the differential system describing all the reactions that occur during nucleated protein polymerization. We denote 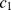 the protein monomeric concentration and 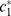 the one of a misfolded monomeric species which displays the ability to polymerize. 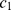 monomers transform into this monomeric species 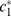 at the rate 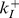, and 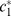 transform back to 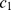 at the rate 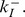



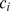
 represents the concentration of polymers made up of 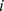 monomers. We assume that polymers and monomers are degraded with a size-dependent degradation rate denoted 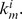 The misfolded monomers 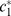 are able to polymerize to give rise to a nucleus 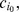 composed of 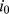 monomeric units, with the rate 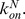 As proposed by Oosawa and co-authors [4], a nucleus is generated by the addition of an object to highly unstable entities that are too transitory to be observed. The object stabilizing the highly unstable entities can be a monomer (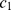). If we consider a nucleus 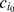 with a size 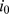, its formation does not consist in a sequential addition of 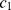 till 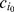 (where it would be represented by 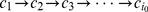), but follows an 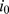 order kinetic (where 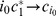).

This nucleus can dissociate at the rate 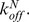 We make the reasonable assumption that there is an equilibrium between monomers and oligomers [4].
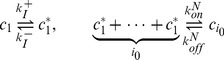
(1)


Polymers of size 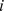 larger than 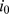 can polymerize or depolymerize, which is the gain or the loss of a single monomeric unit: the elongating species is assumed here to be 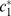 (our model is easy to adapt to other cases, *e.g* if the elongating species is a dimer or an oligomer [8]). Those reactions occur at the rate 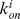 and 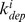 respectively.
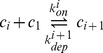
(2)


Polymers can also coalesce with other polymers or break into two smaller polymers. For the sake of simplicity, we assume that a polymer can only break into two pieces at the exact same time (a breakeage into 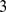 or more pieces is generally much more hazardeous, so that it can be neglected). Coagulation of two polymers of respective size i and j occurs at the rate 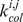. Fragmentation of a polymer of size i gives rise to smaller polymers of size 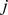 and 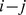 (where 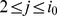), at the rate 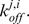


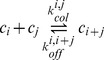
(3)


We could have kept the same notation for fragmentation and depolymerization, by denoting 
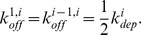
 We prefered however to distinguish them, because they involve reactions of different kinds, so that the orders of magnitude may appear different.

Let us define 
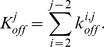
 This represents the total rate with which a polymer of size 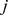 can break to give smaller polymers. By symmetry we have that 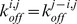 and 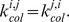


The following model is the exact deterministic transcription of the previously considered reactions. It could be completed by other reactions (polymerization pathways, other types of conformational exchange, for instance) to adapt to any possible case. The variation 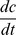 of the species 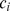 (or 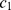, 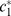) depends on two phenomena: 1) their rates of consumption, including depolymerization into a smaller polymer (or transformation into 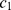 in the case of 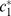), polymerization into a higher polymer (or transformation into 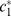 in the case of 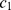) and degradation 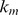, and 2) their rates of production, *i.e.* polymerization from smaller polymer (or transformation from 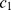 in the case of 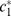) and depolymerization from higher polymer (or transformation from 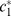 in the case of 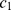). This induces the following equations.
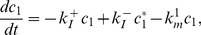
(4)

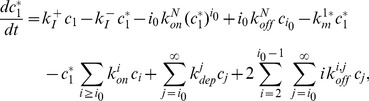
(5)

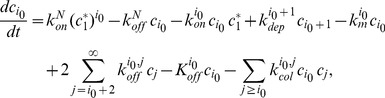
(6)

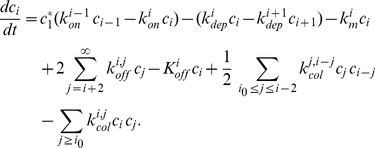
(7)


This model and variants of it have been extensively studied, either in the general case in the mathematical literature (see [10], [14] and references therein), or when applying simplifying assumptions in the biological literature [4], [6]–[8]. It is an efficient tool to study protein aggregation when the average size of protein 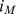 is of a reasonable order. However, for long polymer reactions, this modeling technique may be time-consuming and therefore inefficient to understand the underlying complexity. One can notice the resemblance between this infinite ODE model and a coupled PDE [15].

### From ODEs to PDE: a New Size-structured Model

We propose here a new size-structured model composed of two ODEs and one PDE in the case of a large average size 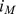 of polymers - *i.e.*, 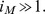 The main idea is to replace the discrete size 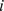 of a polymer by a continuous variable 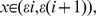 in which we have defined the small parameter 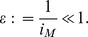 In the same way, the densities 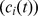 are replaced by a continuous-in-size function 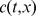 (see supplementary data S1 for more details). This model can be derived from the infinite set of ODEs if the two following assumptions hold.

First, for most polymer sizes 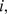 there is only a slight difference between what happens for 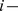 mers and for 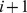-mers. In other terms, even if quantities and reaction rates vary, it occurs in a “continuous” manner, implying only slight changes from one size 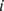 to its neighbor sizes 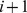 and 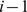 except for a small number of values. For instance, for degradation coefficients 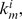 it is formalized as: There exists a constant, denoted below 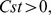 such that
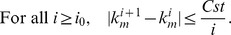



**Figure 1:**
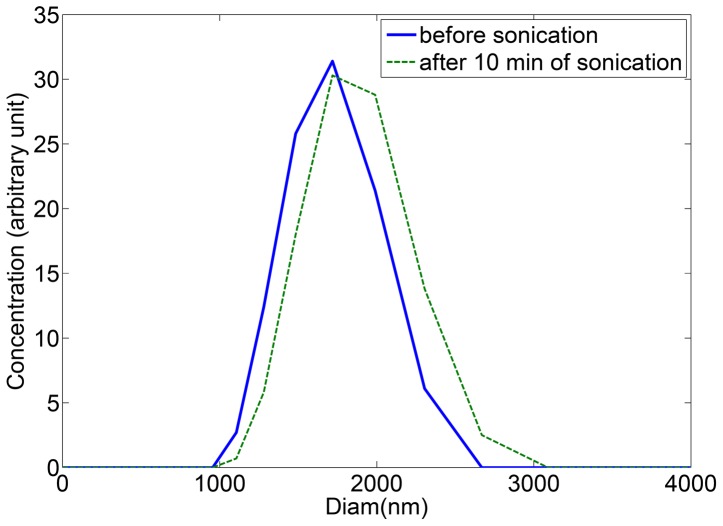
PolyQ41 Fibrils size distribution before (blue plain line) and after (dashed green) 10 min of sonication. The absence of any change in the distribution shows that neither fragmentation nor coalescence occured.

This assertion allows a continuous viewpoint on the equations for 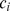. It also means that disruptions in the concentrations or in the coefficients can only appear at some specific points, that will have to be identified, and that are meaningful biologically. Though, this assertion appears to be natural since the conformational changes in polymers only occur at specific sizes [16]. Moreover, having a look at experimental size distributions (Figure 1) confirms how natural it is to view the size of polymers as a continuous quantity.

The second and quite standard assumption is that at the beginning of the reaction, when polymer concentrations remain small compared to monomers, polymerization is the main process, whereas fragmentation and coalescence are secondary processes [4], [6]. This assumption can be replaced if necessary by a similar one, such as the existence of a dominant polymerization by 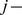
mer addition, with 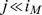 a relatively small oligomer. In such a case, the polymerization terms 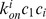 would be replaced by 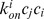 in the equations, and a similar treatment can apply.

We refer to supplementary data S1 for a rigorous mathematical formulation of these two assertions. They are obtained when the system of equations is rescaled, and this allows us to estimate the relative contribution of each process to the overall dynamics.

Let us turn to the nucleus 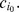 In this equation, the two assertions make it possible to ignore the influence of fragmentation and coalescence. Then as we are in the case where 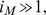 the time-dependency of the equation for 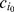 is much faster than the one for 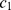: it can be written 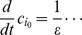 (see supplementary data S1). Hence, it is valid to suppose that it reaches its equilibrium instantaneously, and we can replace Equation (6) by
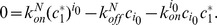



We thus obtain the following equality, which generalizes well-established formulas [6]

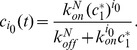
(8)


We can now write the following coupled ODE and PDE system, where 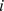 is replaced by a continuous variable 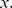 Differences are replaced by derivatives and sums by integrals.
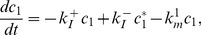
(9)

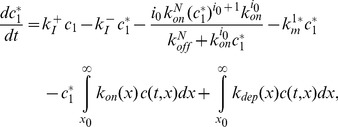
(10)

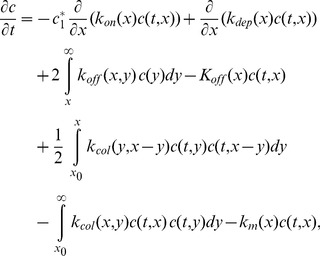
(11)

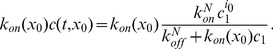
(12)


Complete rigorous mathematical derivation can be found in supplementary data S1, and also shows that generally the third term in the right-hand side of Equation (10) (the ratio 
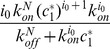
) is negligible. Even mathematical approximation theorems can be written to validate the model, as is done for instance in [10], [11], [17].

The advantages are twofold. First, it allows us to investigate numerically, using standard and well-known numerical schemes (see [18]), how a change in the coefficients can influence the overall reaction, and, more specifically, the size distribution. Also, inverse problem techniques could allow size-dependent parameters to be estimated (see for instance [19], [20]). Secondly, it is easier to handle mathematically. Theoretical analysis can help us understand the intrinsic mechanisms and formulate new paradigms [21], [22].

### Application to PolyQ Polymerization

Aggregation of polyglutamine (PolyQ)-containing proteins is responsible for several neurodegenerative disorders including Huntington’s disease. We have carried out biophysical analyses to investigate the aggregation kinetics of PolyQ41, which are peptides containing a repetition of 41 glutamine residues per monomer. Such a length of PolyQ repetition per molecule is sufficient to induce aggregation *in vitro* and in transfected cells [23].

Due to its simplicity, PolyQ provides an excellent model system to test our mathematical model. According to the experimental observations (Figure 1), fragmentation can be ignored. Indeed, in Figure 1, the size distribution of PolyQ41 fibrils did not change after 10 min of ultrasound treatments, showing that polymer-to-polymer reactions do not occur.

In order to determine whether coalescence occurs, we monitored simultaneously two types of measurements, polymer size and total polymerized mass. Polymer size was estimated by a static light scattering (SLS) signal. SLS is governed by the weighted average mass of oligomers and therefore highly depends on oligomer size. It can be viewed as a measurement of 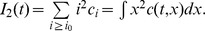 Total polymerized mass was followed by thioflavine T (ThT) fluorescence. Such fluorescence arises from interactions between ThT and the peculiar structure of amyloids, relatively independently of amyloid size (above a certain size threshold). ThT can be mathematically expressed by 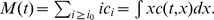 If there were coalescence, the weighted average polymer size would continue to grow even when the total polymerized mass 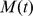 reached a plateau, so the second moment 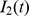 would continue to grow after the plateau has been reached by 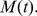 Here, however, both curves reach the plateau roughly simultaneously (see supplementary data S2). Therefore we conclude that coalescence is negligible. As described in Materials and Methods, the spontaneous polymerization of PolyQ41 is prevented by a glutathione s-transferase (GST) tag attached to PolyQ41 peptide. Such experimental system has the advantage of providing a system where only monomeric species are present at time 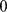, *i.e.* no seeding was required for polymerization: 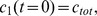 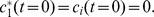 As the GST-polyQ41 does not constitute the pro-aggregative conformer, the PolyQ41 aggregation needs to be ignited by an irreversible enzymatic cleavage (here by thrombin hydrolysis), releasing the GST region apart from PolyQ41. This enzymatic cleavage can be assimilated to an activation process along which the poly Q41 monomer turns into a structurally activated form prone to aggregation. This led us to establish a minimal activation scheme in which the GST-polyQ41, denoted by 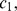 is converted into an active form denoted 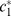 with a constant rate 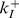. The nucleus size 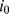, of unknown value, can be equal to 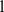, 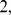 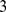 or even more. With these assumptions, Model (4)–(7) becomes
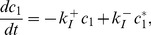
(13)

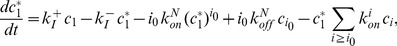
(14)

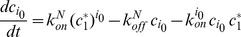
(15)

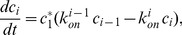
(16)and we use the continuous version of this model, given by (9)–(12), which becomes



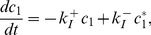
(17)

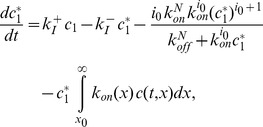
(18)

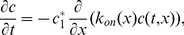
(19)

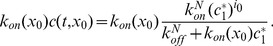
(20)


As an initial approach, we tested piecewise linear polymerization rates. They are linear from 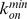 to 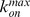 on 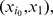 constantly equal to 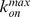 on 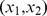 and linearly decreasing to zero on 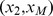 with 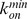 and 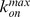 parameters to be calibrated. We arbitrarily set 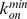 and 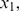 which led to 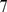 free parameters. We have also tested two different kinds of kinetics when 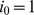: first, the special case where there is no nucleus, *i.e.* the polymerization process starts directly from 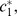 which means 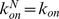 and 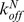 is negligible. This reaction scheme was unable to fit properly even a single experimental curve so we abandoned it. Second, the case when the previous model is unchanged but where 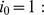 this means that the nucleus 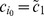 is a monomeric species differing only from 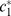 in its conformation. The elongating species remains the intermediate 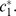 In the following, 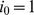 refers to this second case.

**Figure 2:**
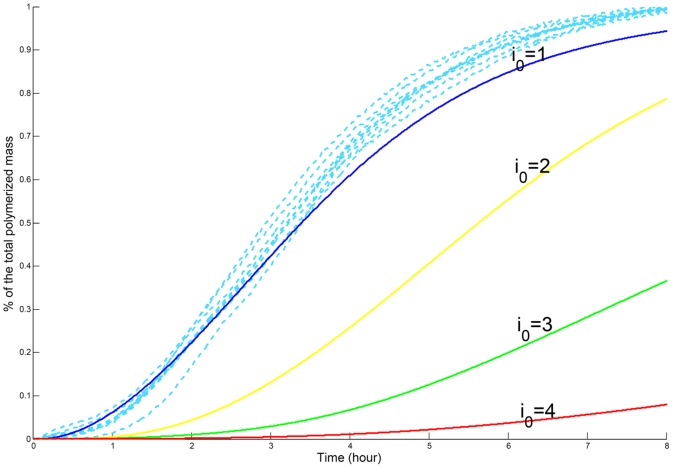
Simulation vs Experiments for Experimental Set 1, for an initial PolyQGST concentration of 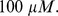. The parameters were first estimated for an experimental set of initial concentration 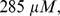 then we compared the experimental measures (dotted lines) for an initial concentration 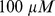 with the simulations (in solid lines) for 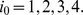 We see that the smaller 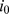 is, the closer the simulation to experimental curves.

The parameters of this model were then estimated by fitting experimental data on PolyQ41 protein polymerization. We performed this in two successive ways. The first consists in fitting separately each experimental curve, corresponding to a given experiment, at a given concentration. The result is that whatever 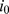 is, the fit is excellent for any curve, with a measurement error from 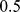 to 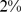 in 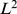 adimensioned norms (see supplementary data S2). It gives almost undistinguishable curves. However, the variability among the optimal coefficients was large, which led us to the second step. This consisted in fitting *simultaneously* all the curves of experiments carried out in identical experimental conditions, but for different concentrations. The global adimensioned error (in 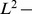
norm) diminished with 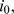 and reached its lowest level for 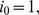 as shown in Figure 2. For larger values of the nucleus, the error is moreover too large for the model to be acceptable (results shown in supplementary data S2). It gives solid ground to the assumption, already suggested in the literature [24], that the nucleus is of size 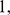 but with a specific and unconventional nucleation-elongation reaction scheme, where the elongating species 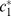 and the nucleus 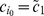 are distinct conformers.

Another result of our simulations is that 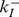 is negligible, thus we can suppose that 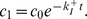 In the same way, we can compare 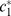 to the solution of the following differential equation
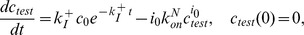

*i.e.,* neglect the contribution of polymers in the equation for 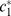: it fits perfectly for the total duration of the lag phase.

### Application to the Knowles et al. Model [7]

As seen for the application to PolyQ, the fully general model (9)–(12) is not yet directly applicable, precisely because of its general character. It can be thought of as the departure point for numerical, biological and mathematical analysis; and it is indeed a powerful way to tackle polymerization issues. To illustrate our approach, we have applied our model to experimental data of amyloid protein aggregation from other authors and we have compared or transposed our model to the recently published models that were accompanying the data [7], [8].

In [7], Knowles and coauthors set up a model for polymerization of breakable filament assembly. This model is an analytical approximation that they have applied to (potential) experimental data and compared to exact equations representing the experimental data. For their approximation model, Knowles and coauthors made the following assumptions.

- Polymerization at a constant rate independent of the size of the polymers,
- no degradation of polymers neither monomers,
- the size of the nucleus is 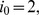

- fragmentation rate depends linearly on the size of the polymer: 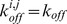 constant,
- no coalescence,
- nucleation disaggregation occurs with the same rate 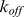 as depolymerization.

**Figure 3:**
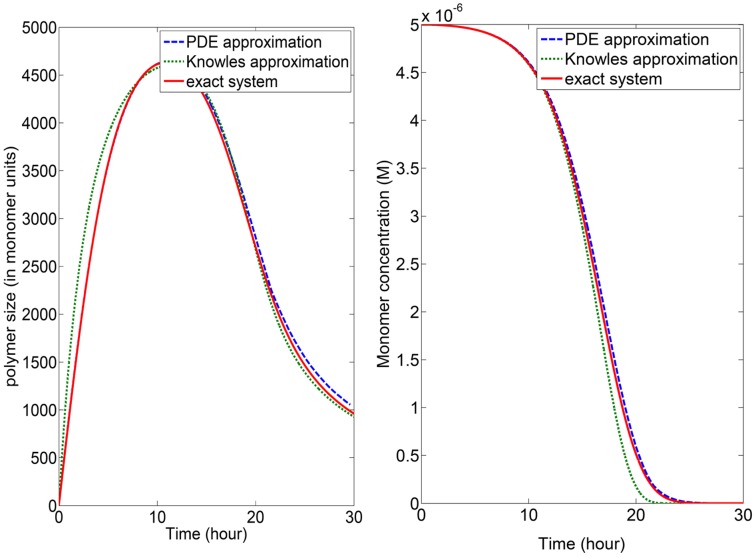
Numerical solution of Equation (21) (22) either using the exact equations, directly representing (potential) experimental data, the PDE approximation, or the analytical approximation proposed in [**7**]. Left: average size of polymers. Right: monomers concentration. It is clear that the PDE approximation gives excellent results. The parameters used for the exact equations (i.e. values for elongation rate, fragmentation rate and nucleus size) are those from Fig. 1 of [7].

With these assumptions, it is well-known that the original ODE system simplifies by summation on a system of 2 non linear coupled ODEs (Equations (3a) and (3b) in [7]), namely:
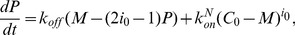
(21)

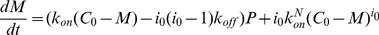
(22)where 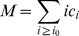 represents the total polymerized mass, and 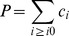 represents the total number of polymers. They approximate this system by an analytical formula, justified by a fixed point method and shown numerically to give a good approximation. To apply our method, we first look at the average size 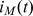 of polymers, which is given by 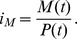 It is shown in Figure 3 for the parameter values 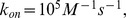 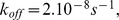 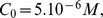 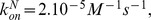 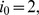 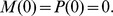 All these values, taken from [7] (fig. 1 of Knowles™ manuscript), directly represent the exact system of (potential) experimental data. We see that our assertion of large polymers is satisfied. Similarly, we check that the range of parameters that they proposed fit to our other assumptions, so that our method can be applied. The assumption on 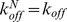 implies that the nucleus dissociation term in the equations for 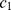 and 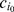 is negligible: indeed we have 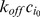 to be compared to 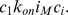


We followed their modelling ideas but our method allows us to relax their assumptions in the following sense.

- Polymerization is not necessarily constant, but values 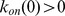 for small polymers of size 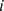 close to 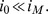

- We neglect degradation of small polymers and of monomers, but we keep a degradation for large polymers,
- *I*_0_ = 2,
- fragmentation rate does not necessarily depend linearly on the size of the polymer, but it is true for small polymers: 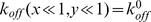 constant,
- coalescence is negligible compared to polymerization as long as 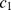 remains in the order of 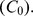

With these assumptions, System (9)–(12) can be simplified as follows:
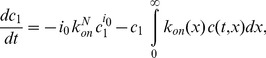
(23)

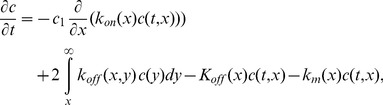
(24)

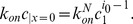
(25)


**Figure 4:**
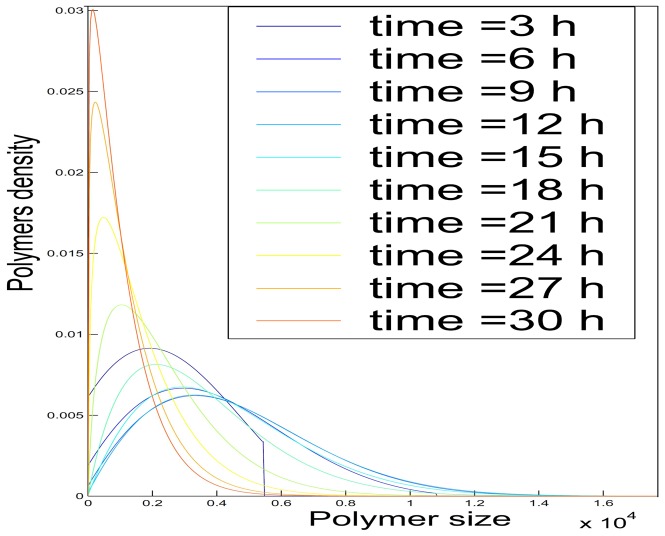
Adimensionned Distributions of the sizes of polymers for various times. To obtain adimensionned distributions of polymer sizes, our model was applied to data taken from Figure 1 of [7]. From 6 to 18 hours one can see that the distribution remains roughly stable.

If we take as in [7] 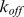 and 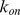 constant, we recover System (??)(??) by summation, but with the terms 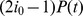 and 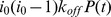 neglected. Numerical simulations are shown in Figure 3, and we see that this simplification allows a perfect fit with the complete model, fast simulations, and a better understanding of which reaction dominates at any moment (since we have access to size distributions, see Figure 4).

#### Comments on Size Distributions

For the size parameters taken from [7], fig. 1, we are able to observe the evolution of polymer size distributions over time: see Figure 4. At the beginning of the reaction (in this particular case, for a time between 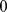 and 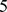 hours), the average size increases very fast. Then it reaches an equilibrium, and between 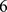 to 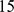 hours it reaches an exponential regime during which the whole size distribution, not only the average size, is quite steady. An explanation for this could be taken from [25] for instance. After this period, the average size decreases - and ultimately, the model shows that 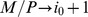 but this would be accomplished only after a very long period of time. A good test for the model proposed by [7] would be to check whether size distribution of polymers resembles such a one-peak distribution. If not, the assumptions would have to be relaxed, *e.g.* by taking variable coefficients [25].

### PDE Model Applied to the Xue et al. Model [8]

Xue and colleagues present a new strategy to analyse the self-assembly of misfolded proteins into amyloid fibrils [8]. They analysed fibril length distribution of 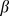
2-microglobulin, a protein involved in dialysis-related amyloidosis. Xue and colleagues have developed the following approach. Based on a large data set of experimental growth curves, transitional general parameters of the time-curve, namely the length of the lag phase (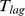) and the slope (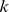) of the reaction curve at the inflexion point were extracted. Several theoretical models are simulated using the ODE formulation and the theoretical transitional parameters 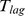 and 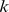 were extracted from the numerical growth curve in the same way as for the experimental curve (see Table S2 in Supplementary data S3). Then the best model and its parametrization were determined by comparing the theoretical values with the experimental data through least-squares analysis. This powerful approach is based on the simulation of a full ODE system (with one equation per size of aggregates) for each model investigated and no simplifications were made to reduce the dimension of this system. As a consequence, the method is time-consuming, which limits the number of mechanisms studied and the maximal polymer size (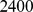 in [8]). In addition, estimation of the best fitting model is based only on general parameters of the curve, which do not seem much sensitive to the distribution of the fragmentation process (see supplementary data S3). To overcome these limitations, we propose transposing their approach using PDE models, allowing for i) faster simulations, ii) no limitation in the size of aggregates, and iii) development of inverse problem techniques ([26], [27]) to estimate parameters using the overall time evolution process.

Xue *et al* investigated 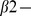
microglobulin growth, using models including different processes: a pre-polymerization step (characterized by either no pre-polymerization, or monomer-dimer equilibrium and dimer addition mechanism, or conformation exchange), an elongation of the aggregates following a one-step function, a linear function or a power function, and a possible secondary process such as fragmentation. Their best-fit model is given by:

- No conformational exchange, no coalescence and no degradation of polymers or monomers,
- the size of the nucleus is 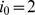 and nucleus dissociation occurs only through depolymerization,
- polymerization and depolymerization follow a one-step function with the step at 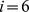,
- fragmentation into two smaller polymers occurs.

Thus, using the previously introduced notations, the original ODE system can be written
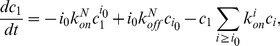
(26)

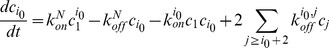
(27)

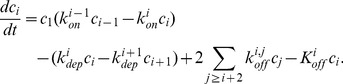
(28)


For the particular choice of fragmentation made in [8], however, fragmentation in polymers of size 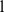 is close to 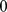. This ODE system is then formally equivalent to the following PDE system:
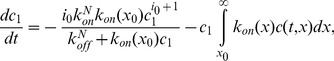
(29)

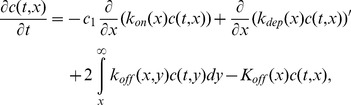
(30)

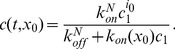
(31)


Due to the shape of the polymerization process, which has a step at 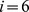 (meaning that 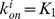 for 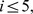 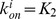 for 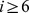), if the step is high, that is if 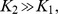 it is however preferable to keep all the ODEs occurring for 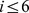 and to set up the PDE (30) only for 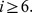 We then adapt the boundary condition (31) as shown in supplementary data S3. This can also be approximated by the Bishop and Ferrone model [6] by adjusting a nucleus critical size to 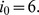 Similar work can be done for the different processes studied in [8]. Our study allowed us to enhance their approach by quick investigation of different fragmentation kernels, showing that the shape of the fragmentation does not influence the polymerization dynamics (see supplementary data S3).

## Discussion

We proposed a new model (9)–(12) to serve as a global framework to investigate the leading mechanisms of nucleation-elongation processes in amyloid fibrils’ assemblies. We applied it to PolyQ41 aggregation, demonstrating experimentally that coalescence and fragmentation were negligible, and predicting by our simulations that the monomer activation was irreversible. Moreover, it highlighted the early step of PolyQ41 nucleus formation and assemblies. With regard to the bibliography, the concept of nucleus in protein aggregation remains obscure. Here the analysis of PolyQ polymerization suggested a kinetic scheme in which 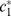 is at an equilibrium with 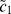. These two species are monomeric and only differ in their conformation. According to the conventional model of nucleation-elongation process [4], the nucleus is thermodynamically stabilized by the addition of at least one monomer. Here we proposed an unconventional mechanism of nucleation in which the 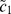 formation constitutes the limiting step in the polymerization process which is stabilized by an interaction with 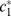. Therefore, the formation of the 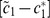 complex constitutes the first proaggregative species. Furthermore, during the formation of this complex, a structural information exchange should occur between 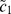 and 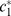. To reach the formation of a nucleus, two changes of conformation are hence required. The first one arises from the GST-cleavage of 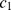 to a conformer 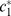 released as a random coil structure, that is not proaggregative. The second change of conformation is an internal change of the random coil 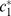 into a proaggregative species 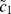 that is still monomeric.

Our approach also proved highly efficient when applied to previously designed models [7], [8], where it can be adapted and used to pursue the research further. We believe it could be applied to many other cases, providing both a unified framework and an efficient way to carry out fast simulations, model discrimination [28], inverse problem methods and analysis.

## Materials and Methods

### Model Derivation

To derive the continuous model, we first write a rescaled version of the model, that makes use of typical orders of magnitude. Then, quantifying our assumptions, we approximate sums by integrals and differences by derivatives. Finally, from the equation for 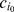 we deduce the boundary condition for 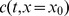 (full details in supplementary data S1).

### Numerical Implementation

To avoid useless conversions, we implemented the PDE model (9)–(12) with dimensioned numbers, and checked *a posteriori* that the considered orders of magnitude fit the assumptions. We use an explicit upwind scheme - finer methods can be used such as WENO [18].

### Parameter Estimation

The parameter estimation was performed by a least-square approach. For 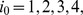 we searched for the optimal set of parameters such that it minimized the quadratic distance between the data points obtained by ThT measures and the simulated curve of the mass, represented by 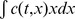 in the PDE model or by 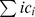 in the ODE one. The minimization task was performed by the CMAES algorithm [29]. It was run with 50 different initial parameters sets. Then the optimal solution was used as an initial guess and the minimization algorithm was run again 50 times.

### Experimental Results

#### GST-PolyQ production

The GST-Q41 expression vector was described by Masino et al [30]. GST-polyQ41 fusion protein was produced in E.Coli BL21DE3 and purified by affinity chromatography using Glutathione Sepharose affinity beads (Pharmacia).

#### Fragmentation experiments

The Fragmentation experiments were performed using an immersion sonotrod oscillating at 40 kHz. The size distributions of polyQ fibrils were monitored before and after sonication by dynamic light scattering (DLS, Wyatt).

#### Kinetic experiments

All polymerization experiments were performed at 33°C. Aggregation was initiated by thrombin addition (0.5 unit/ÂµM of GST-PolyQ41) leading to the release of PolyQ41 peptide from GST. The aggregation was monitored either by Thioflavine T (Tht) (100 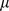
M) in a 96-well plate fluorescence spectrometer or by a homemade multiwavelength static light scattering/fluorescence system (SLS).

## Supporting Information

Figure S1
**Parameter estimation considering each curve separately.** Time evolution of PolyQ41 polymerized mass for an initial PolyQGST concentration equal to 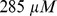. The experimental results are plotted in dotted line and the best-fit curve in solide line. 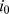 is set to 3. Best-fit parameters are 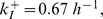 *
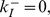

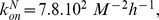

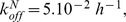

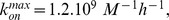

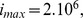

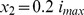
.*
(TIF)

Figure S2
**Parameter estimation for Experimental Set 1 when 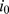 is set to 3.** Time evolution of the adimensioned PolyQ41 polymerized mass for an initial PolyQGST concentration equal to 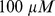 (A), 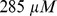 (B), 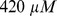
(C). Dotted curves represent experimental results. The solid curve is the best-fit. The global error in 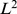 adimensioned norm was equal to 40% and the optimal parameters are very close to those of Figure 1.(TIF)

Figure S3
**Parameter estimation for Experimental Set 1 when 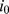 is set to 1.** Time evolution of the adimensioned PolyQ41 polymerized mass for an initial PolyQGST concentration equal to 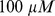 (A), 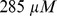 (B), 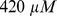 (C). Dotted curves represent experimental results. The solid curve is the best-fit. The global error in in 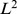 adimensioned norm was equal to 11%. The best-fit parameters are 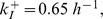 *
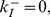

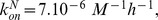

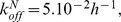

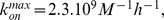

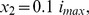

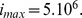
*
(TIF)

Figure S4 Left: Size distribution of the fragmentation rate for an aggregation of size 20, following a uniform distribution (black) or a mechanical-based distribution (red) of fragmentation. Right: Simulated normalized reaction progress curves of amyloid formation for a uniform distribution (black) and a mechanical-based distribution (red) of fragmentation. See below for the numerical values.
(TIF)

Figure S5 **Examples of simulated size distribution of the aggregates for a uniform distribution (black) and a mechanical-based distribution (red) of fragmentation.** See above for the numerical values.
(TIF)

Supplementary Data S1 **Model derivation from ODE to PDE.**
(PDF)

Supplementary Data S2 **Application to PolyQ41 polymerization.**
(PDF)

Supplementary Data S3 **Effect of the fragmentation distribution on the kinetics of the Xue et al. model** [**1**] **.**
(PDF)
