# Supplementary figures and images for: An Efficient Kinetic Model for Assemblies of Amyloid Fibrils and Its Application to Polyglutamine Aggregation
Source: PLoS One. 2012 Nov 13;7(11):e43273. doi: 10.1371/journal.pone.0043273 (PMC3496744; doi:10.1371/journal.pone.0043273)

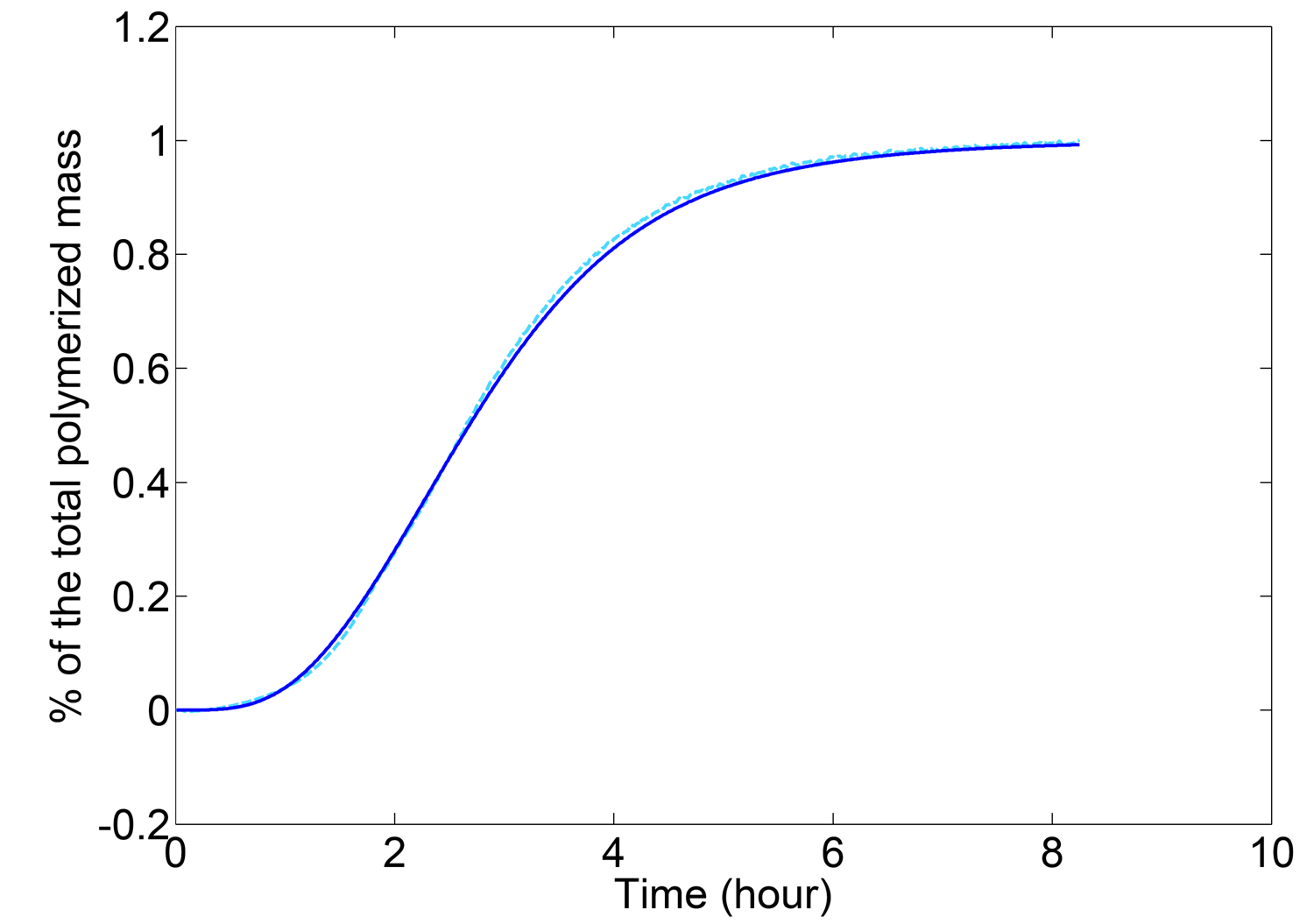

Supplement: Figure S1 — Parameter estimation considering each curve separately. Time evolution of PolyQ41 polymerized mass for an initial PolyQGST concentration equal to . The experimental results are plotted in dotted line and the best-fit curve in solide line. is set to 3. Best-fit parameters are . (TIF) [file pone.0043273.s001.tif]

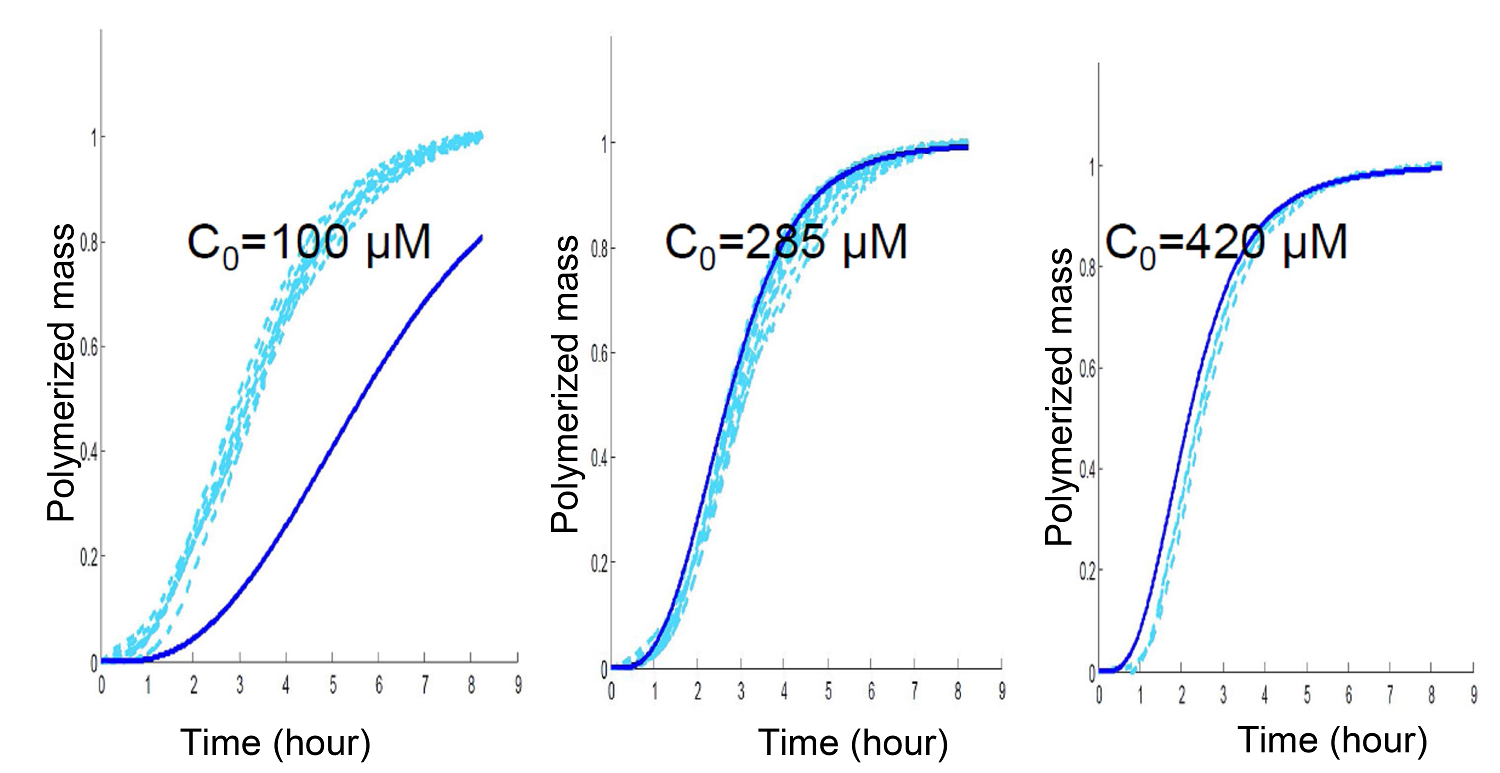

Supplement: Figure S2 — Parameter estimation for Experimental Set 1 when is set to 3. Time evolution of the adimensioned PolyQ41 polymerized mass for an initial PolyQGST concentration equal to (A), (B), (C). Dotted curves represent experimental results. The solid curve is the best-fit. The global error in adimensioned norm was equal to 40% and the optimal parameters are very close to those of Figure 1. (TIF) [file pone.0043273.s002.tif]

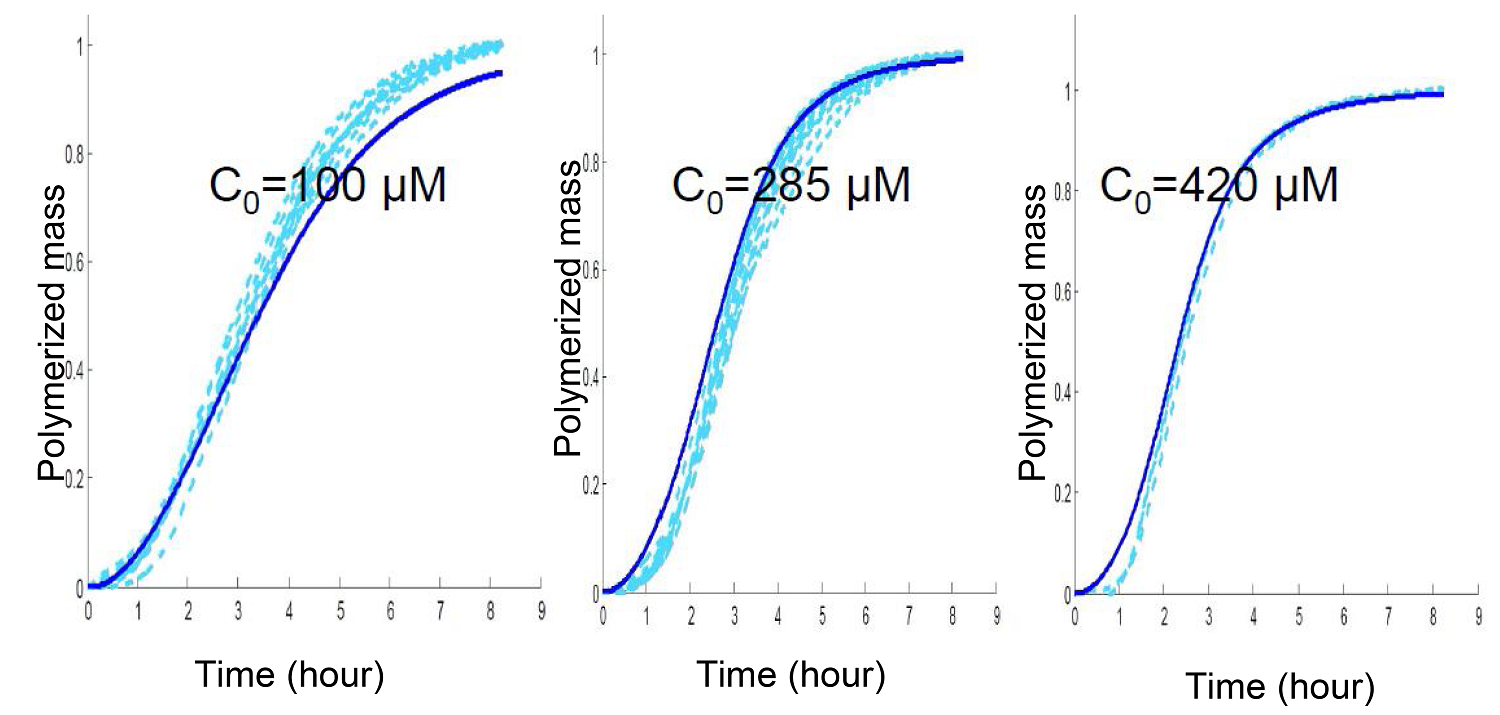

Supplement: Figure S3 — Parameter estimation for Experimental Set 1 when is set to 1. Time evolution of the adimensioned PolyQ41 polymerized mass for an initial PolyQGST concentration equal to (A), (B), (C). Dotted curves represent experimental results. The solid curve is the best-fit. The global error in in adimensioned norm was equal to 11%. The best-fit parameters are (TIF) [file pone.0043273.s003.tif]

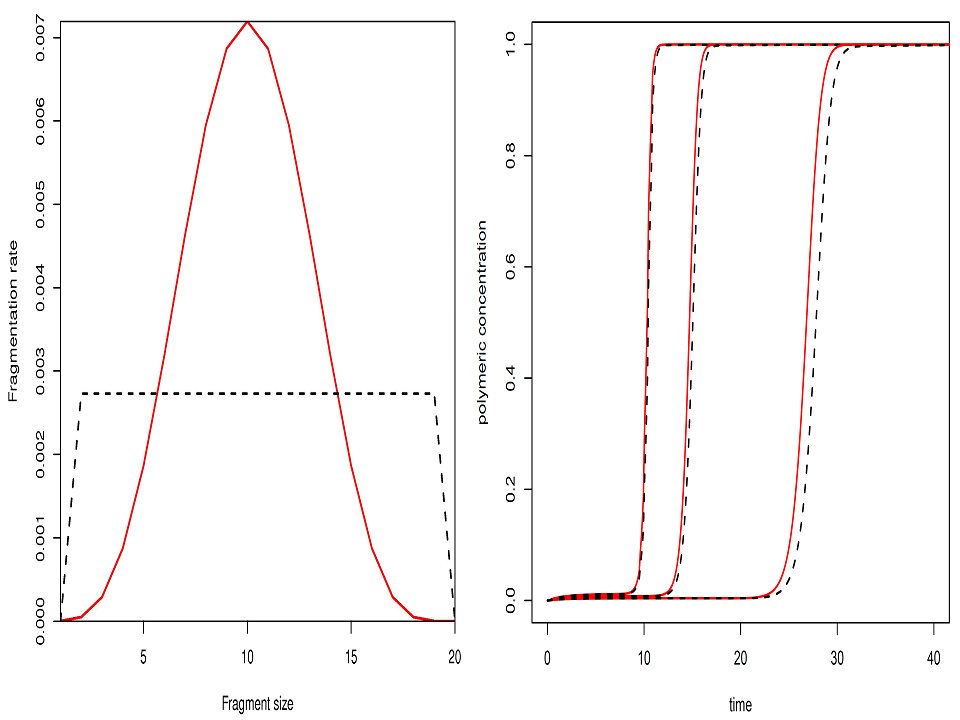

Supplement: Figure S4 — Left: Size distribution of the fragmentation rate for an aggregation of size 20, following a uniform distribution (black) or a mechanical-based distribution (red) of fragmentation. Right: Simulated normalized reaction progress curves of amyloid formation for a uniform distribution (black) and a mechanical-based distribution (red) of fragmentation. See below for the numerical values. (TIF) [file pone.0043273.s004.tif]

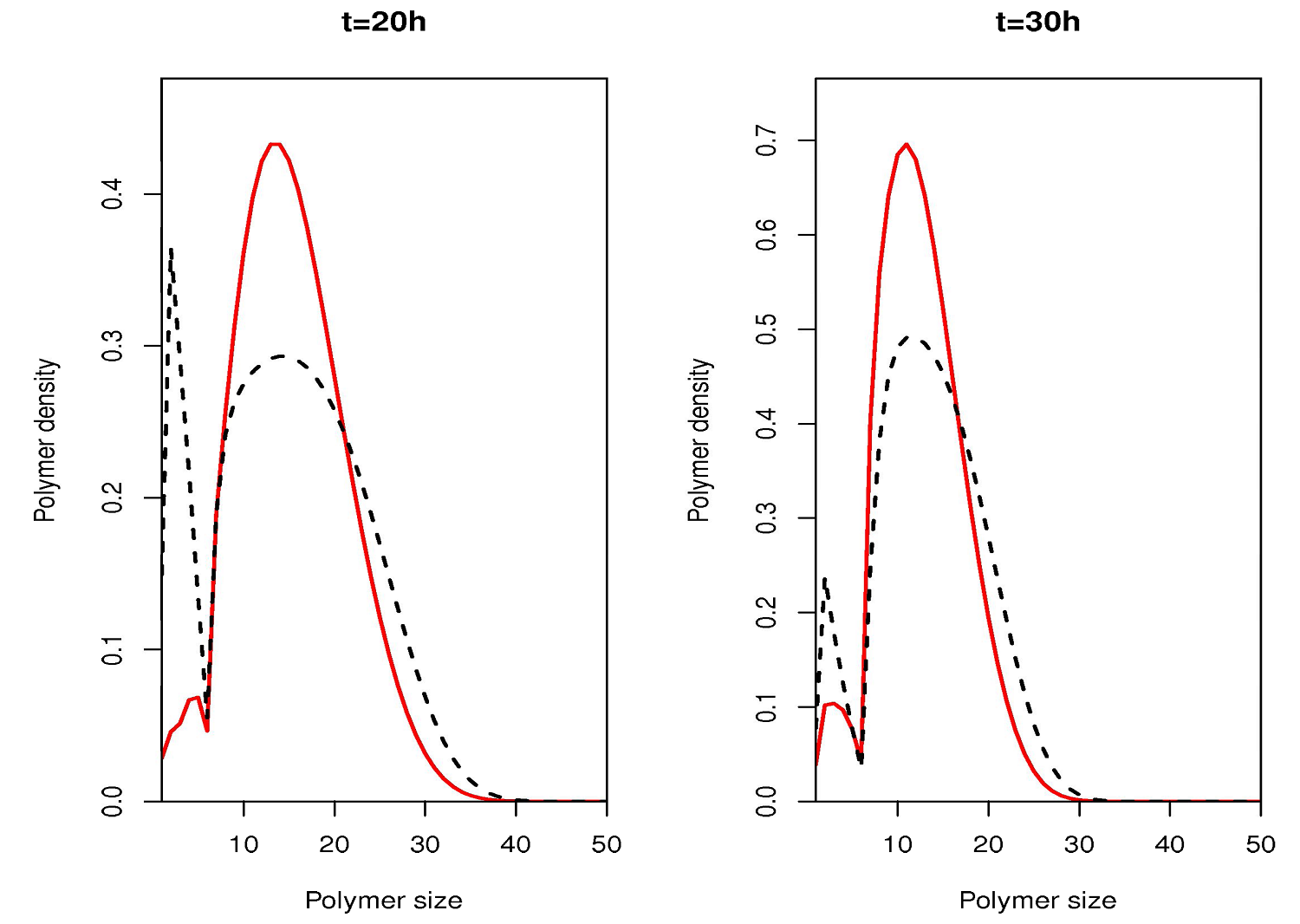

Supplement: Figure S5 — Examples of simulated size distribution of the aggregates for a uniform distribution (black) and a mechanical-based distribution (red) of fragmentation. See above for the numerical values. (TIF) [file pone.0043273.s005.tif]
