# Supplementary material for: An Efficient Kinetic Model for Assemblies of Amyloid Fibrils and Its Application to Polyglutamine Aggregation
Source: PLoS One. 2012 Nov 13;7(11):e43273. doi: 10.1371/journal.pone.0043273 (PMC3496744; doi:10.1371/journal.pone.0043273)
Supplement: Supplementary Data S1 — Model derivation from ODE to PDE. (PDF) [file pone.0043273.s006.pdf]

Supporting Information of the article : An Efficient Kinetic Model for Assemblies of Amyloid Fibrils and Its Application to Polyglutamine Aggregation

Supplementary Data S1: Model Derivation from ODE to PDE

In this Supplementary Information, we detail all the formal calculations that lead to the new PDE model proposed in the main article. For the sake of completeness, in a first section we give all the notations and assumptions, together with the original infinite ODE model. We also recall how simplifying assumptions lead to a close system of at most 5 equations: this is a generalization of what has been used in many previous articles [1, 2].

In Section 2, we derive the PDE model. The initial step consists in writing a rescaled system. Under assumptions on the relative orders of magnitude, we then derive the PDE model, and obtain the boundary condition. Numerical tests show excellent fits between the discrete and the continuous model (see for instance in the main text when the model is applied to Knowles et al. model [2]).

1 A General ODE Model for Protein Polymerization

1.1 Biological assumptions

We denote c_1 the protein monomeric concentration, c_1^* a monomeric conformer of the protein, and c_i the concentration of polymers of size i. We consider the following reactions.

1. Monomers can exchange with the conformer compartment, with rates k_I^+ and k_I^-.
2. Monomer conformers are able to polymerize to give rise to a nucleus c_{i_0}, composed of i_0 monomers, with rates k_{on}^N and k_{off}^N. We have used here already derived simplifications (see [1] for instance) that allow us to condense the chain reaction leading to the nucleus into this unique reaction ; different cases may of course be considered in the same way.
3. Polymers or monomers can be degraded, each with a specific degradation rate denoted k_m^i.
4. Polymers of size i larger than i_0 can either depolymerize, *i.e.* lose one single monomer splitted into one monomer plus one polymer of size $i-1$, with a rate k_{dep}^i; or fragmentate to give rise to smaller polymers of size $j \leq i-2$ and $i-j$ with a rate $k_{off}^{j,i}$; or polymerize by monomer addition with a rate k_{on}^i; or yet coalesce with other polymers of size j with a rate $k_{col}^{i,j}$. We could have kept the same notation for fragmentation and depolymerization, by denoting $k_{off}^{1,i} = k_{off}^{i-1,i} = \frac{1}{2}k_{dep}^i$. We prefered however to distinguish them, because they involve reactions of different kinds, so that the orders of magnitude may appear very different. We will discuss further this point when doing the PDE approximation.
5. Polymers of size smaller to i_0 are unstable, and are immediately degraded into monomers.
6. Polymers can only split into two smaller fragments (not simultaneously into three or more fragments).

Though this model already seems very complete, it is not completely universal: for instance, we neglected the following possible reactions.

- Polymers of size i all behave in the same way : this excludes the consideration of polymerization pathways.
- There is no conformer of any species except monomers, especially we did not consider conformational exchange for the nucleus.

These assumptions could be relaxed and the original system complexified or adapted if necessary ; as it is however, it is sufficient to serve as a proof of concept for our method.

1.2 ODE Model

Let us define

$$K_{off}^{j} = \sum_{i=2}^{j-2} k_{off}^{i,j}.$$

This represents the total rate with which a polymer of size j can fragmentate to give smaller polymers. By symetry we have

$$k_{off}^{i,j} = k_{off}^{j-i,j}, \qquad k_{col}^{i,j} = k_{col}^{j,i}.$$

The following model is the exact deterministic transcription of the previously considered reactions. It could be completed by other reactions (polymerization pathways, other types of conformational exchange for instance).

$$\frac{dc_1}{dt} = -k_I^+ c_1 + k_I^- c_1^* - k_m^{(1)} c_1, \tag{1}$$

$$\begin{aligned} \frac{dc_1^*}{dt} &= k_I^+ c_1 - k_I^- c_1^* - i_0\, k_{on}^N\, (c_1^*)^{i_0} + i_0\, k_{off}^N\, c_{i_0} - k_m^{1*} c_1^* \\ &\quad - c_1^* \sum_{i \ge i_0} k_{on}^i\, c_i + \sum_{j=i_0}^{\infty} k_{dep}^j c_j + 2 \sum_{i=2}^{i_0-1} \sum_{j=i_0}^{\infty} i\, k_{off}^{i,j}\, c_j, \end{aligned} \tag{2}$$

$$\begin{aligned} \frac{dc_{i_0}}{dt} &= k_{on}^N\, (c_1^*)^{i_0} - k_{off}^N\, c_{i_0} - k_{on}^{i_0}\, c_{i_0}\, c_1^* + k_{dep}^{i_0+1} c_{i_0+1} - k_m^{i_0} c_{i_0} \\ &\quad + 2 \sum_{j=i_0+2}^{\infty} k_{off}^{i_0,j}\, c_j - K_{off}^{i_0} c_{i_0} - \sum_{j \ge i_0} k_{col}^{i_0,j}\, c_{i_0}\, c_j, \end{aligned} \tag{3}$$

$$\begin{aligned} \frac{dc_i}{dt} &= c_1^* (k_{on}^{i-1}\, c_{i-1} - k_{on}^i\, c_i) - (k_{dep}^i c_i - k_{dep}^{i+1} c_{i+1}) - k_m^i c_i \\ &\quad + 2 \sum_{j=i+2}^{\infty} k_{off}^{i,j}\, c_j - K_{off}^i c_i + \frac{1}{2} \sum_{i_0 \le j \le i-i_0} k_{col}^{j,i-j}\, c_j\, c_{i-j} - \sum_{j \ge i_0} k_{col}^{i,j} c_i\, c_j. \end{aligned} \tag{4}$$

Existence and uniqueness of a solution for this kind of model was investigated by [3, 4] for instance. A fundamental property is mass balance: summing-up all the reactions, and multiplying the $i-$mer concentration by i (*i.e.* by its mass expressed in terms of number of monomers), we obtain

$$\frac{d}{dt}\left(c_1 + c_1^* + \sum_{i=i_0}^{\infty} i c_i \right) = -k_m^{(1)} c_1 - k_m^{1*} c_1^* - \sum_{i \ge i_0} i k_m^i c_i. \tag{5}$$

This means that the total mass can only change either by production (absent here) or degradation, but polymerization, fragmentation and coalescence do not change it.

Similarly, we can define $P(t) = \sum_{i\geq i_0} c_i$ the total number of polymers, and write

$$\begin{aligned}\frac{dP}{dt} &= k_{on}^N\,(c_1^*)^{i_0} - k_{off}^N\,c_{i_0} - \sum_{i\geq i_0} k_m^i c_i \\ &+ \sum_{i=i_0}^{\infty} K_{off}^i\,c_i - \frac{1}{2}\sum_{j\geq i_0}\sum_{i\geq i_0} k_{col}^{i,j} c_i\,c_j.\end{aligned} \tag{6}$$

This means that polymerization and depolymerization do not change the total number of polymers, only nucleation, fragmentation and coalescence do.

These two properties are fundamental, and any approximating model must keep them.

1.3 Simplification by drastic assumptions

To simplify this infinite set of ODEs, the most common way consists in using the previously seen equations for polymers mass and quantity (5), (6), and making very simplifying assumptions to write a closed system involving only c_1, c_1^*, c_{i_0}, P and M. This has been made for instance by Masel *et al.* in [5] for Prion equations, by Knowles *et al* in [2] for breakable filament assembly, and in a huge number of other work. Let us make the following (very) simplifying assumptions.

1. Polymerization occurs through a linear process, *i.e.*

$$\exists\, K_{on}^{(1)},\; K_{on}^{(2)} \in \mathbb{R},\, K_{on}^{(1)} + i_0 K_{on}^{(2)} \geq 0, \quad \forall\, i \geq i_0, \qquad k_{on}^i = K_{on}^{(1)} + iK_{on}^{(2)}. \tag{7}$$

   With this relation, in Equation (2) we have: $c_1^* \sum_{i\geq i_0} k_{on}^i\,c_i = K_{on}^{(1)} c_1^* P + K_{on}^{(2)} c_1^* M$.

2. Depolymerization occurs through a linear process, *i.e.*

$$\exists\, K_{dep}^{(1)},\; K_{dep}^{(2)} \in \mathbb{R},\, K_{dep}^{(1)} + i_0 K_{dep}^{(2)} \geq 0, \quad \forall\, i \geq i_0, \qquad k_{dep}^i = K_{dep}^{(1)} + iK_{dep}^{(2)}. \tag{8}$$

   Similarly, it makes it possible to write in Equation (2): $\sum_{j=i_0}^{\infty} k_{dep}^j c_j = K_{dep}^{(1)} P + K_{dep}^{(2)} M$.

3. Degradation is constant *i.e.*

$$\exists\, K_m \geq 0, \quad \forall\, i \geq i_0, \qquad k_m^i = K_m \tag{9}$$

   Hence, we have $\sum_{j=i_0}^{\infty} k_m^j c_j = K_m P$ and $\sum_{j=i_0}^{\infty} j k_m^j c_j = K_m M$.

4. Fragmentation occurs through an homogeneous process, *i.e.*

$$\exists\, K_{off} \geq 0, \quad \forall\, 2 \leq i \leq j-2,\, j \geq i_0, \quad k_{off}^{ij} = K_{off}. \tag{10}$$

   This means that each node of each polymer behaves in the same way, having the same probability to break. In Equation (2) this implies

$$2\sum_{i=2}^{i_0-1}\sum_{j=i_0}^{\infty} i\,k_{off}^{i,j}\,c_j = 2K_{off}\sum_{j=i_0}^{\infty} c_j \sum_{i=2}^{min(i_0-1,j-2)} i = K_{off}\Big(\big(i_0(i_0-1)-2\big)P - 2(i_0-1)c_{i_0}\Big),$$

   and in Equation (6) to

$$\sum_{j=i_0}^{\infty} K_{off}^j\,c_j = \sum_{j=i_0}^{\infty}\sum_{i=2}^{j-2} k_{off}^{ij}\,c_j = K_{off}\sum_{j=i_0}^{\infty}(j-3)c_j = K_{off}(M-3P).$$

5. Coalescence occurs through an homogeneous and linear process, *i.e.*

$$\exists\, K_{col}^{(1)},\ K_{col}^{(2)} \in \mathbb{R},\ K_{col}^{(1)} + 2i_0 K_{col}^{(2)} \geq 0, \quad \forall\, i,\, j \geq i_0, \qquad k_{col}^{ij} = K_{col}^{(1)} + (i+j)K_{col}^{(2)}. \tag{11}$$

In Equation (6) this leads to

$$\frac{1}{2}\sum_{j\geq i_0}\sum_{i\geq i_0} k_{col}^{i,j} c_i\, c_j = \frac{1}{2}\sum_{j\geq i_0}\sum_{i\geq i_0} K_{col}^{(1)} c_i\, c_j + \sum_{j\geq i_0}\sum_{i\geq i_0} K_{col}^{(2)} i c_i\, c_j = \frac{1}{2}K_{col}^{(1)}P^2 + K_{col}^{(2)}MP.$$

6. In the equation for c_{i_0}, we neglect the depolymerization term involving c_{i_0+1} and assume that $\sum\limits_{j=i_0+2}^{\infty} K_{off} c_j \approx \sum\limits_{j=i_0+1}^{\infty} K_{off} c_j$. This last assumption will be justified later, and is much weaker than the previous ones (it only involves orders of magnitude and not specific rates values). Moreover, in the case where $K_{dep} = K_{off}$ (for instance as in [2, 5]) it is unnecessary.

Altogether, these assumptions lead to the following closed system:

$$\frac{dc_1}{dt} = -k_I^+ c_1 + k_I^- c_1^* - k_m^{(1)} c_1, \tag{12}$$

$$\begin{aligned}\frac{dc_1^*}{dt} &= k_I^+ c_1 - k_I^- c_1^* - i_0\, k_{on}^N\, (c_1^*)^{i_0} + i_0\, k_{off}^N\, c_{i_0} - k_m^{1*} c_1^* - c_1^*(K_{on}^{(1)} P + K_{on}^{(2)} M) \\ &\quad + K_{dep}^{(1)} P + K_{dep}^{(2)} M + K_{off}\Big(\big(i_0(i_0-1)-2\big)P - 2(i_0-1)c_{i_0}\Big),\end{aligned} \tag{13}$$

$$\begin{aligned}\frac{dc_{i_0}}{dt} &= k_{on}^N\, (c_1^*)^{i_0} - k_{off}^N\, c_{i_0} - k_{on}^{i_0}\, c_{i_0}\, c_1^* - k_m^{i_0} c_{i_0} + 2K_{off}P \\ &\quad - (i_0-1)K_{off} c_{i_0} - c_{i_0}\Big((K_{col}^{(1)} + i_0 K_{col}^{(2)})P + K_{col}^{(2)} M\Big),\end{aligned} \tag{14}$$

$$\frac{dP}{dt} = k_{on}^N\, (c_1^*)^{i_0} - k_{off}^N\, c_{i_0} - K_m P + K_{off}(M - 3P) - \frac{1}{2}K_{col}^{(1)} P^2 - K_{col}^{(2)} MP, \tag{15}$$

$$\frac{dM}{dt} = -\frac{d}{dt}\Big(c_1 + c_1^*\Big) - k_m^{(1)} c_1 - k_m^{1*} c_1^* - K_m M. \tag{16}$$

2 From the ODE to the PDE Model

The resemblance between the infinite ODE model (1)–(4) and a coupled PDE [6, 7] is obvious, simply by replacing sums by integrals and differences by derivatives. The intuitive idea is the following: since the average size of a polymer is very large, let us consider that we have a *continuum* of sizes.

To link such a continuous-in-size model with the ODE model (1)–(4), supplementary assumptions on the orders of magnitude of the different parameters are needed. Such assumptions provide a deeper understanding of how each process contributes to the reaction. Our method is based on such a PDE approximation, that reveals both very informative and much simpler for numerical simulation. However, the orders of magnitude must be carefully checked, and the ODE should not be translated into a simplified PDE in the same way at any moment of the reaction.

The steps are the following.

First, we write Model (1)–(4) in a properly rescaled version.

Second, we introduce a small parameter ε, justify its use, and build a grid. We also give a precise mathematical definition to each of the three "fundamental assertions" given in the main text.

Finally, we derive the PDE model.

2.1 Rescaled Model

We introduce the following characteristic quantities.

- $\mathcal{V}$ and $\mathcal{V}^*$ the characteristic values respectively for the concentration of monomers c_1 and c_1^*,
- $\mathcal{C}_{i_0}$ and $\mathcal{C}$ the respective characteristic values for the concentration of polymers c_{i_0} and c_i,
- $\mathcal{K}_+$ and $\mathcal{K}_-$ the respective chararacteristic values for the exchange rates k_I^+ and k_I^-,
- $\mathcal{K}_{on}^N$ and $\mathcal{K}_{off}^N$ the respective characteristic values for the (de)nucleation rates k_{on}^N and k_{off}^N,
- $\mathcal{K}_{on}$ and $\mathcal{K}_{dep}$ the respective characteristic values for the (de)polymerisation frequencies k_{on}^i and k_{dep}^i,
- $\mathcal{K}_m$ the characteristic value for the sedimentation frequencies of polymers k_m^i,
- $\mathcal{K}_{off}$ the characteristic value for the fragmentation frequencies of polymers $k_{off}^{i,j}$,
- $\mathcal{K}_{col}$ the characteristic value for the coalescence frequencies of polymers $k_{col}^{i,j}$.

The rescaled quantities are defined by

$$\bar c_1(t) = \frac{c_1(t)}{\mathcal{V}}, \quad \bar c_1^*(t) = \frac{c_1^*(t)}{\mathcal{V}^*}, \quad \bar c_{i_0}(\bar t) = \frac{c_{i_0}(t)}{\mathcal{C}_{i_0}}, \quad \bar c_i(t) = \frac{c_i(t)}{\mathcal{C}} \quad \text{for} \quad i > i_0,$$

$$\bar k_I^+ = \tfrac{k_I^+}{\mathcal{K}_+}, \qquad \bar k_I^- = \tfrac{k_I^-}{\mathcal{K}_-}, \qquad \bar k_{on}^N = \frac{k_{on}^N}{\mathcal{K}_{on}^N}, \qquad \bar k_{off}^N = \frac{k_{off}^N}{\mathcal{K}_{off}^N},$$

$$\bar k_{on}^i = \frac{k_{on}^i}{\mathcal{K}_{on}}, \qquad \bar k_{off}^{ij} = \frac{k_{off}^{ij}}{\mathcal{K}_{off}}, \quad \bar k_{dep}^i = \frac{k_{dep}^i}{\mathcal{K}_{dep}}, \qquad \bar k_m^i = \frac{k_m^i}{\mathcal{K}_m}, \qquad \bar k_{col}^{ij} = \frac{k_{col}^{ij}}{\mathcal{K}_{col}}.$$

Equations (1)–(4) become :

$$\frac{d\bar c_1}{dt} = -\mathcal{K}_+ \bar k_I^+ \bar c_1 + \frac{\mathcal{K}_- \mathcal{V}^*}{\mathcal{V}} \bar k_I^- \bar c_1^* - \mathcal{K}_m \bar k_m^{(1)} \bar c_1, \tag{17}$$

$$\begin{aligned}\frac{d\bar c_1^*}{dt} &= \frac{\mathcal{K}_+ \mathcal{V}}{\mathcal{V}^*} \bar k_I^+ \bar c_1 - \mathcal{K}_- \bar k_I^- \bar c_1^* - i_0\, \mathcal{K}_{on}^N (\mathcal{V}^*)^{i_0-1} \bar k_{on}^N\, (\bar c_1^*)^{i_0} + i_0\, \frac{\mathcal{K}_{off}^N \mathcal{C}_{i_0}}{\mathcal{V}^*}\, \bar k_{off}^N\, \bar c_{i_0} \\ &\quad - \mathcal{K}_{on} \mathcal{C} \bar c_1^* \sum_{i \ge i_0} \bar k_{on}^i\, \bar c_i - \mathcal{K}_m \bar k_m^{1*} \bar c_1^* + 2 \frac{\mathcal{K}_{off} \mathcal{C}}{\mathcal{V}^*} \sum_{i=1}^{i_0-1} \sum_{j=i_0}^{\infty} i\, \bar k_{off}^{i,j}\, \bar c_j + \frac{\mathcal{K}_{dep} \mathcal{C}}{\mathcal{V}^*} \sum_{j=i_0}^{\infty} \bar k_{dep}^j \bar c_j,\end{aligned} \tag{18}$$

$$\begin{aligned}\frac{d\bar c_{i_0}}{dt} &= \frac{\mathcal{K}_{on}^N \mathcal{V}^{*i_0}}{\mathcal{C}_{i0}} \bar k_{on}^N\, (\bar c_1^*)^{i_0} - \mathcal{K}_{off}^N \bar k_{off}^N\, \bar c_{i_0} - \mathcal{K}_{on} \mathcal{V}^* \bar k_{on}^{i_0}\, \bar c_{i_0}\, \bar c_1^* + \mathcal{K}_{dep} \frac{\mathcal{C}}{\mathcal{C}_{i_0}} \bar k_{dep}^{i+1} \bar c_{i+1} \\ &\quad + 2 \frac{\mathcal{K}_{off} \mathcal{C}}{\mathcal{C}_{i_0}} \sum_{j=i_0+1}^{\infty} \bar k_{off}^{i_0,j}\, \bar c_j - \mathcal{K}_{off} \bar K_{off}^{i_0} c_{i_0} - \mathcal{K}_{col} \mathcal{C} \sum_{j \ge i_0} \bar k_{col}^{i_0,j} \bar c_{i_0}\, \bar c_j - \mathcal{K}_m \bar k_m^{i_0} \bar c_{i_0},\end{aligned} \tag{19}$$

$$\begin{aligned}\frac{d\bar c_i}{dt} &= \mathcal{V}^* \mathcal{K}_{on} \bar c_1^* (\bar k_{on}^{i-1}\, \bar c_{i-1} - \bar k_{on}^i\, \bar c_i) + \mathcal{K}_{dep} (\bar k_{dep}^{i+1} \bar c_{i+1} - \bar k_{dep}^i \bar c_i) - \mathcal{K}_m \bar k_m^i \bar c_i \\ &\quad + \mathcal{K}_{off} \left(2 \sum_{j=i+1}^{\infty} \bar k_{off}^{i,j}\, \bar c_j - \sum_{j=2}^{i-2} \bar k_{off}^{j,i} \bar c_i \right) + \mathcal{K}_{col} \mathcal{C} \left(\frac{1}{2} \sum_{i_0 \le j < i} \bar k_{col}^{j,i-j} \bar c_j\, \bar c_{i-j} - \sum_{j \ge i_0} \bar k_{col}^{i,j} \bar c_i\, \bar c_j \right).\end{aligned} \tag{20}$$

Let us also give the mass-balance equation in this rescaled setting:

$$\frac{d}{dt} \left(\bar c_1 + \frac{\mathcal{V}^*}{\mathcal{V}} \bar c_1^* + \frac{\mathcal{C}_{i0}}{\mathcal{V}} i_0 \bar c_{i_0} + \frac{\mathcal{C}}{\mathcal{V}} \sum_{i=i_0+1}^{\infty} i \bar c_i \right) = -\mathcal{K}_m \left(\bar k_m^{(1)} \bar c_1 + \bar k_m^{1*} \bar c_1^* + \sum_{i \ge i_0} i \bar k_m^i \bar c_i \right). \tag{21}$$

2.2 Main Assertions

By the use of comparison in the orders of magnitude, we derived a continuous model from this infinite set of ODEs. To do so, we first introduced the average size i_M of polymers.

First of all, as stated in the article, we place ourselves in the case where **the average size i_M of a polymer is very large - *i.e.*, much larger than** 1. This can be formalized mathematically as

$$\varepsilon := \frac{1}{i_M} \ll 1. \tag{22}$$

Now, we know how to approximate a discrete size by a continuous one: we define $x_0^\varepsilon = \varepsilon i_0$, (what is almost zero if $i_0 \ll i_M$), $x_i^\varepsilon = \varepsilon i$ (what is in the order of 1 if $i \sim i_M$) and

$$c_\varepsilon(t,x) := \sum_{i=i_0}^{\infty} \bar{c}_i(t)\, \chi_{\{[x_i^\varepsilon, x_{i+1}^\varepsilon[\}}(x), \tag{23}$$

where $\chi_{\{I\}}$ is the Heaviside function on the interval I.

We defined similarly functions on the real line for each family of parameters: $k_{on}^\varepsilon(x)$ for $\bar{k}_{on}^i$, $k_{col}^\varepsilon(x,y)$ and $k_{off}^\varepsilon(x,y)$ respectively for $\bar{k}_{off}^{i,j}$ and $\bar{k}_{col}^{i,j}$, $k_{dep}^\varepsilon(x)$ for $\bar{k}_{dep}^i$. For a family of parameters P^i, the function P^ε is defined on each interval $[x_i^\varepsilon, x_{i+1}^\varepsilon[$ by the value of P^i.

We then assumed in the article that ***for most polymer sizes i, there is only a slight difference between what happens for $i-$mers and for $i+1$-mers.*** Mathematically, this means that these piecewise functions tend, when ε vanishes, to regular functions. The following assumption is thus the exact statement of such an assumption (see for instance [6, 8] for more details, derived for less general models):

$$\begin{gathered}\text{For each family of parameters } P^i \text{ appearing in the model (17)–(20),}\\ \exists K \geq 0, \forall\, i \geq i_0, \qquad P^i \leq K, \qquad |P^{i+1} - P^i| \leq \tfrac{K}{i}.\end{gathered} \tag{24}$$

Concerning the fragmentation rates $\bar{k}_{off}^{ij}$, the condition is (see [8]):

$$\begin{gathered}\text{For the parameters } \bar{k}_{off}^{ij}\ ,\\ \exists K \geq 0, \forall\, j \geq i_0, \qquad \bar{k}_{off}^{ij} \leq \tfrac{K}{j}, \qquad |\sum_{i' \leq i} \bar{k}_{off}^{i'j+1} - \bar{k}_{off}^{i'j}| \leq \tfrac{K}{j}.\end{gathered} \tag{25}$$

Though there is no explicit condition yet published for the coagulation coefficients $\bar{k}_{col}^{ij}$, we can conjecture from [8, 9] that we need a variant of Assumption (25). Under such assumptions, it is possible to prove that when ε vanishes, the function P_ε defined as c_ε by (23) converges (up to a subsequence) toward a continuous function $P(x)$ and k_{off}^{ij} converges to $k_{off}(x,y)$ in a proper way (see Lemmas 9 and 10 in [8]). This is a kind of "continuity assumption". Indeed discontinuities may appear, for some given polymer sizes. Such cases could also be taken into account in our model: the derivation proposed here would only have to be adapted to a different context.

Let us now look at the mass balance (21). What we expect is that, during the main part of the experiment, monomer mass, conformers mass and the polymerized mass $\sum\limits_{i \geq i_0} i\bar{c}_i$ live within the same order of magnitude. We can compare the orders of magnitude, and find that the following relation is necessary:

$$\frac{\mathcal{C}}{\mathcal{V}} \sum_{i=i_0+1}^{\infty} i\bar{c}_i \sim \frac{\mathcal{C}}{\mathcal{V}} \sum_{i=i_0}^{i_M} i \sim \frac{\mathcal{C}}{\mathcal{V}} i_M^2 \sim \frac{\mathcal{C}}{\mathcal{V}} \frac{1}{\varepsilon^2} \sim 1,$$

hence we have that

$$\frac{\mathcal{C}}{\mathcal{V}} = \varepsilon^2.$$

With the previous definitions, we can formally write

$$\frac{\mathcal{C}}{\mathcal{V}} \sum_{i=i_0+1}^{\infty} i\bar{c}_i = \sum_{i=i_0+1}^{\infty} \int_{\varepsilon i}^{\varepsilon(i+1)} x_i \bar{c}_i dx \approx \int_{x_0=\varepsilon i_0}^{\infty} x c_\varepsilon(t,x) dx.$$

The symbol $\approx$ here means that under some mathematical assumptions as in [6], the infinite sum tends to the integral sum when ε vanishes. In the same way, taking also $\frac{\mathcal{V}}{\mathcal{V}^*} = \mathcal{K}_m = 1$, and $\mathcal{C}_{i_0} \leq \mathcal{C}$ so that the term with $\bar{c}_{i_0}$ becomes negligible compared to $\sum\limits_{i \geq i_0+1} i\bar{c}_i$, the mass balance (21) becomes

$$\frac{d}{dt}\Big(c_1 + c_1^* + \int_{x_0}^{\infty} x c(t,x) dx\Big) = -\Big(k_m^{(1)} \bar{c}_1 + k_m^{1*} c_1^* + \int_{x_0}^{\infty} x k_m(x) c(t,x) dx\Big). \tag{26}$$

We treat similarly each contribution in the equations, first focusing on sums and differences. Sums are interpreted as integrals, differences as approximations of derivatives, and we identify the piecewise functions with their regular limit functions. Equation (17) first leads to

$$\mathcal{K}_+ = \mathcal{K}_- \frac{\mathcal{V}^*}{\mathcal{V}} = \mathcal{K}_m = 1,$$

so that Equation (1) remains unchanged in the continuous model. Note that if this is not verified, *e.g.* if monomers are very quickly transformed in conformers, leading to neglect the transconformation step, the model would be easily adapted. In Equation (18), we write

$$\mathcal{K}_{on}\mathcal{C} \sum_{i \geq i_0} \bar{k}_{on}^i \, \bar{c}_i = \frac{\mathcal{K}_{on}\mathcal{C}}{\varepsilon} \int_{x_0}^{\infty} k_{on}^{\varepsilon}(x) c_\varepsilon(t,x) dx,$$

and we need, to have a contribution of order 1 in the equation, to have that $\mathcal{K}_{on}\mathcal{C} = \varepsilon$. Similarly, still in Equation (18),

$$\frac{\mathcal{K}_{dep}\mathcal{C}}{\mathcal{V}^*} \sum_{i \geq i_0} \bar{k}_{dep}^i \, \bar{c}_i = \frac{\mathcal{K}_{dep}\mathcal{C}}{\varepsilon \mathcal{V}^*} \int_{x_0}^{\infty} k_{dep}^{\varepsilon}(x) c_\varepsilon(t,x) dx,$$

what leads to $\frac{\mathcal{K}_{dep}\mathcal{C}}{\mathcal{V}^*} = \varepsilon$. Looking now at Equation (20), we have, still for the polymerization and depolymerization terms

$$\mathcal{V}^* \mathcal{K}_{on} (\bar{k}_{on}^{i-1} \, \bar{c}_{i-1} - \bar{k}_{on}^i \, \bar{c}_i) \approx \mathcal{V}^* \mathcal{K}_{on} \varepsilon \frac{\partial}{\partial x}\big(k_{on}(x) c(t,x)\big),$$

which leads to the assumption that $\mathcal{V}^*\mathcal{K}_{on} = \frac{1}{\varepsilon}$. Together with the previously seen relation $\mathcal{K}_{on}\mathcal{C} = \varepsilon$, this implies

$$\frac{\mathcal{C}}{\mathcal{V}^*} = \frac{\mathcal{C}}{\mathcal{V}} = \varepsilon^2. \tag{27}$$

This is very logical: indeed, what is comparable to monomers concentration c_1 or c_1^* is not an isolated polymer concentration c_i, but the total concentration of polymerized monomers, *i.e.* $\sum\limits_{i=i_0}^{\infty} i c_i$, whose order of magnitude is $i_M^2 \mathcal{C}$.

For the fragmentation terms, in Equation (20) we aim at writing

$$\mathcal{K}_{off}\Big(2\sum_{j=i+1}^{\infty}\bar{k}_{off}^{i,j}\,\bar{c}_j-\sum_{j=2}^{i-2}\bar{k}_{off}^{j,i}\bar{c}_i\Big)\approx\frac{\mathcal{K}_{off}}{\varepsilon}\Big(2\int_{x_i}^{\infty}k_{off}(x,y)c(t,y)dy-(\int_{0}^{x_i}k_{off}(y,x)dy)c(t,x)\Big),$$

what leads to $\mathcal{K}_{off}=\varepsilon$. Similarly for the coalescence terms:

$$\mathcal{K}_{col}\mathcal{C}\Big(\frac{1}{2}\sum_{i_0\le j<i}\bar{k}_{col}^{j,i-j}\bar{c}_j\,\bar{c}_{i-j}\,-\sum_{j\ge i_0}\bar{k}_{col}^{i,j}\bar{c}_i\,\bar{c}_j\Big)\approx$$

$$\frac{\mathcal{K}_{col}\mathcal{C}}{\varepsilon}\Big(\frac{1}{2}\int_{x_0}^{x}k_{col}(y,x-y)c(t,y)c(t,x-y)dy-\int_{x_0}^{\infty}k_{col}(x,y)c(t,x)c(t,y)dy\Big),$$

what leads to $\mathcal{K}_{col}\mathcal{C}=\varepsilon$.

These three definitions of $\mathcal{K}_{on}$, $\mathcal{K}_{col}$ and $\mathcal{K}_{off}$ give a precise mathematical definition for the second assumption of the article, stated as ***The polymerization process is the dominant reaction, that is, the fragmentation and the coalescence processes contribute to the reaction at most in a comparable way to the polymerization process, at least at the beginning of the reaction.*** Indeed, the typical value for polymerization process is $\mathcal{K}_{on}\mathcal{V}^*=\varepsilon^{-1}$, whereas the typical orders for coalescence and fragmentation are $\mathcal{K}_{col}\mathcal{C}=\mathcal{K}_{off}=\varepsilon$.

We sum-up all these assumptions by the following relations:

$$\mathcal{K}_{col}\mathcal{C}=\mathcal{K}_{off}=\varepsilon,\qquad\mathcal{V}^*\mathcal{K}_{on}=\frac{1}{\varepsilon}.\tag{28}$$

2.3 PDE Model

Let us now give the final model we are lead to. We already have the mass balance equation (26), and an unchanged equation (17) for c_1. Omitting the overlines, with the previously seen assumptions (22), (24), (25), (27) and (28), Equation (20) leads to

$$\begin{aligned}\frac{\partial c(t,x)}{\partial t}&=-c_1^*\frac{\partial}{\partial x}\big(k_{on}(x)\,c(t,x)\big)+\frac{\partial}{\partial x}\big(k_{dep}(x)c(t,x)\big)-k_m(x)c(t,x)\\&+2\int_{x}^{\infty}k_{off}(x,y)c(t,y)dy-K_{off}(x)c(t,x)\\&+\frac{1}{2}\int_{x_0}^{x}k_{col}(y,x-y)c(t,y)c(t,x-y)dy-\int_{x_0}^{\infty}k_{col}(x,y)c(t,x)c(t,y)dy,\end{aligned}\tag{29}$$

where we have defined $K_{off}(x):=\int_{x_0}^{x}k_{off}(y,x)dy$.

2.4 Boundary Condition and treatment for c_{i_0}

We now have to deal with the most delicate point: how to treat the terms involving c_{i_0}, in Equation (18) as well as in its own equation (19) ? There is also the issue of the boundary condition $c(t,x=x_0)$: indeed, for the PDE problem to be well-posed, we do not need only equations for c_1, c_1^* and $c(t,x)$ but also a boundary condition $c(t,x=x_0)$ replacing the equation for c_{i_0}.

We can find this boundary condition by two different ways. The first more intuitive way consists in treating the exchange terms with k_{off}^N and k_{on}^N appearing in Equations (18) and (19). In order to get finite contributions in Equation (18), we need to set

$$a = \mathcal{K}_{on}^N (\mathcal{V}^*)^{i_0-1} = O_\varepsilon(1), \qquad b = \mathcal{K}_{off}^N \frac{\mathcal{C}_{i_0}}{\mathcal{V}^*} = O_\varepsilon(1),$$

and we can choose $a = b = 1$. In the case of irreversible polymerization we will take $\bar{k}_{off}^N = 0$; in the case of no spontaneous fibrillization (as for the original prion model of [5]) we will take $\bar{k}_{on}^N = 0$. Denoting $\delta = \frac{\mathcal{C}_{i_0}}{\mathcal{C}}$ and using the previously seen orders of magnitude, we have in Equation (19)

$$\frac{d\bar{c}_{i_0}}{dt} = \frac{1}{\varepsilon^2\delta}\bar{k}_{on}^N\,(\bar{c}_1^*)^{i_0} - \frac{1}{\varepsilon^2\delta}\bar{k}_{off}^N\,\bar{c}_{i_0} - \frac{1}{\varepsilon}k_{on}(x_0)\,\bar{c}_{i_0}\,\bar{c}_1^* + \cdots,$$

the $\cdots$ expressing terms of orders of magnitude negligible compared to ε^{-1} or to $\frac{1}{\varepsilon^2\delta}$. This implies that the three first reaction terms in the equation for c_{i_0} occur at a speed much higher than for the other terms of the equation. Thus, we can assume that instantaneous equilibrium is reached, what leads to the following boundary condition (we omit the overlines)

$$c(t,x_0) = \bar{c}_{i_0} = \frac{k_{on}^N c_1^{*i_0}}{k_{off}^N + \varepsilon\delta k_{on}(x_0)c_1^*}. \tag{30}$$

Depending on the ratio δ and on the value of k_{off}^N, this boundary condition can simplify either in $c(t,x_0) = \bar{c}_{i_0} = \frac{k_{on}^N c_1^{*i_0}}{k_{off}^N}$ (see [10]) or in $c(t,x_0) = \bar{c}_{i_0} = \frac{k_{on}^N c_1^{*(i_0-1)}}{\varepsilon\delta k_{on}(x_0)}$. In this last case, the quantity $\mathcal{C}_{i_0}$ is expected to be much larger than $\mathcal{C}_i$, what can be checked numerically. This does not necessarily contradict the fact that the nucleus c_{i_0} is a transient species, whose concentration is very low, remaining almost undetectable: indeed, $\mathcal{C}_i$ may be much lower still, but when i becomes very large, since what is observed is the mass of polymerised monomers of typical size $i_M\mathcal{C}_i$, it becomes detectable.

Introducing this relation in the equation for c_1^* (18), we obtain:

$$\begin{aligned}\frac{dc_1^*}{dt} &= k_I^+ c_1 - k_I^- c_1^* - i_0\,k_{on}^N\,(\bar{c}_1^*)^{i_0+1}\frac{\varepsilon\delta k_{on}(x_0)}{k_{off}^N + \varepsilon\delta k_{on}(x_0)c_1^*} \\ &\quad - c_1^*\int_{x_0}^{\infty} k_{on}(x)c(t,x)dx - k_m^{1*}c_1^* + \int_{x_0}^{\infty} k_{dep}(x)c(t,x)dx.\end{aligned} \tag{31}$$

A second possible way of obtaining formally the boundary condition (30) is to impose both the mass balance equation (26) and the differential equation (31). Integrating (formally) the PDE equation (29) against the weight x, and integration by parts, leads to Equation (30) multiplied by x_0.

Altogether, Equations (1), (29),(31) and (30) is a well-posed PDE system approximating System (1)–(4) under Assumptions (22), (24), (25), (27) and (28). A complete approximation theorem, in the spirit of [8], can be established with the use of moments estimates leading to compactness. For the sake of simplicity, we chose not to give it here and prefered to give only the formal derivation, sufficient to give the intuition.

2.5 Numerical Implementation

In order to avoid useless conversions, we chose to implement directly the PDE model (1), (29)–(31) with dimensioned numbers, and to check *a posteriori* that the considered orders of magnitude fit the mathematical assumptions (22), (24), (25), (27), (28). We use an explicit upwind scheme ; finer methods can be used such as WENO, see [11].

References

1. Oosawa F, Asakura S (1975) Thermodynamics of the polymerization of protein. Academic Press.

2. Knowles TPJ, Waudby CA, Devlin GL, Cohen SIA, Aguzzi A, et al. (2009) An Analytical Solution to the Kinetics of Breakable Filament Assembly. Science 326: 1533-1537.

3. Ball JM, Carr J (1990) The discrete coagulation-fragmentation equations: existence, uniqueness, and density conservation. J Statist Phys 61: 203–234.

4. Ball JM, Carr J, Penrose O (1986) The Becker-Döring cluster equations: basic properties and asymptotic behaviour of solutions. Comm Math Phys 104: 657–692.

5. Masel J, Jansen V, Nowak M (1999) Quantifying the kinetic parameters of prion replication. Biophysical Chemistry 77: 139 - 152.

6. Collet JF, Goudon T, Poupaud F, Vasseur A (2002) The Becker–Döring system and its Lifshitz–Slyozov limit. SIAM J on Appl Math 62: 1488-1500.

7. Pruss J, Pujo-Menjouet L, Webb G, Zacher R (2006) Analysis of a model for the dynamics of prion. Dis Cont Dyn Sys Ser B 6: 225–235.

8. Doumic M, Goudon T, Lepoutre T (2009) Scaling limit of a discrete prion dynamics model. Communications in Mathematical Sciences 7: 839–865.

9. Laurençot P, Mischler S (2002) From the discrete to the continuous coagulation-fragmentation equations. Proc Roy Soc Edinburgh Sect A 132: 1219–1248.

10. Bishop M, Ferrone F (1984) Kinetics of nucleation-controlled polymerization. a perturbation treatment for use with a secondary pathway. Biophysical Journal 46: 631 - 644.

11. Gabriel P, Tine LM (2010) High-order WENO scheme for polymerization-type equations. ESAIM Proc 30: 54–70.
