# Supplementary material for: An Efficient Kinetic Model for Assemblies of Amyloid Fibrils and Its Application to Polyglutamine Aggregation
Source: PLoS One. 2012 Nov 13;7(11):e43273. doi: 10.1371/journal.pone.0043273 (PMC3496744; doi:10.1371/journal.pone.0043273)
Supplement: Supplementary Data S2 — Application to PolyQ41 polymerization. (PDF) [file pone.0043273.s007.pdf]

Supporting Information of the article : An Efficient Kinetic Model for Assemblies of Amyloid Fibrils and Its Application to Polyglutamine Aggregation

Supplementary data S2: Application to PolyQ41 polymerization

Parameters of the PDE model [9]-[12] were estimated using experimental data on PolyQ41 polymerization measured by ThT. Two independent sets of experiments were carried out, using two different devices. The PolyQGST initial concentration ranged from $20\mu M$ to $420\mu M$. For experimental set 1, there were 9 curves for the concentration 100 μM, 13 for 285 μM and 3 for 420 μM. For experimental set 2, there were one curve for each concentration 20, 30, 40, 50 and 80 μM.

We first tested our model by considering each experimental curve separetely and thus computing a parameter set for each of them (see Main Text). We set $k_{on}^{min} = 1h^{-1}M^{-1}$, $x_1 = 0.1i_{max}$. An example of this parameter estimation is shown in Figure S1 for an initial PolyQGST concentration equal to $100\mu M$, in which i_0 was set to 3. For any value of i_0 among 1, 2, 3 or 4, the fit was excellent for all curves: in a least-square approach, we computed the error in L^2 adimensioned norm, calculated as $\frac{||f^{mes}-f^{sim}||_{L^2}}{||f^{mes}||_{L^2}}$, where $||f||_{L^2} = \sqrt{\sum_i f_i^2}$, the values f_i being measured values at time t_i for the notation f^{mes} and simulated values at time t_i for the notation f^{sim}. These errors ranged from 0.5% to 2%.

Secondly, we tested our model by fitting all curves of a given set of experiments simultaneously. Best-fits for experimental set 1 are drawn in Figures S2 and S3, respectively for $i_0 = 3$ and $i_0 = 1$. Global estimation errors for the two experimental sets ranged from 5% to 61% and decreased with i_0 value (Table S1).

The larger i_0 is, the more the lag-time changes with the concentration. Moreover, this dependence is too strong as soon as $i_0 \geq 2$. Indeed, for the lower concentrations, the simulation is too slow compared to experimental data, whereas it is too fast for the higher one, as shown for $i_0 = 3$ in Figure S2.

Figure Legends

Figure S 1. Parameter estimation considering each curve separately. Time evolution of PolyQ41 polymerized mass for an initial PolyQGST concentration equal to $285\mu M$. The experimental results are plotted in dotted line and the best-fit curve in solide line. i_0 is set to 3. Best-fit parameters are $k_I^+ = 0.67h^{-1}$, $k_I^- = 0$, $k_{on}^N = 7.8.10^2 M^{-2}h^{-1}$, $k_{off}^N = 5.10^{-2}h^{-1}$, $k_{on}^{max} = 1.2.10^9 M^{-1}h^{-1}$, $i_{max} = 2.10^6$, $x_2 = 0.2i_{max}$.

Figure S 2. Parameter estimation for Experimental Set 1 when i_0 is set to 3. Time evolution of the adimensioned PolyQ41 polymerized mass for an initial PolyQGST concentration equal to $100\mu M$ (A), $285\mu M$ (B), $420\mu M$(C). Dotted curves represent experimental results. The solid curve is the best-fit. The global error in L^2 adimensioned norm was equal to 40% and the optimal parameters are very close to those of Figure 1.

Figure S 3. Parameter estimation for Experimental Set 1 when i_0 is set to 1. Time evolution of the adimensioned PolyQ41 polymerized mass for an initial PolyQGST concentration equal to $100\mu M$ (A), $285\mu M$ (B), $420\mu M$(C). Dotted curves represent experimental results. The solid curve is the best-fit. The global error in in L^2 adimensioned norm was equal to 11%. The best-fit parameters are $k_I^+ = 0.65h^{-1}$, $k_I^- = 0$, $k_{on}^N = 7.10^{-6}M^{-1}h^{-1}$, $k_{off}^N = 5.10^{-2}h^{-1}$, $k_{on}^{max} = 2.3.10^9 M^{-1}h^{-1}$, $x_2 = 0.1i_{max}$, $i_{max} = 5.10^6$.

Tables

Table S 1. Optimization results

i_0	Minimal error (adimensioned L^2 norm)	
	First set of experiments	Second set of experiments
1	11%	5%
2	37%	7%
3	44%	9%
4	59%	29%
