# Supplementary material for: An Efficient Kinetic Model for Assemblies of Amyloid Fibrils and Its Application to Polyglutamine Aggregation
Source: PLoS One. 2012 Nov 13;7(11):e43273. doi: 10.1371/journal.pone.0043273 (PMC3496744; doi:10.1371/journal.pone.0043273)
Supplement: Supplementary Data S3 — Effect of the fragmentation distribution on the kinetics of the Xue et al. model [1] . (PDF) [file pone.0043273.s008.pdf]

Supporting Information of the article : An Efficient Kinetic Model for Assemblies of Amyloid Fibrils and Its Application to Polyglutamine Aggregation

Supplementary Data S3: Effect of the fragmentation distribution on the kinetics of the Xue *et al.* model [1]

Xue *et al* investigated $\beta 2-$microglobulin growth, using models including different processes: a pre-polymerization step (characterized by either no pre-polymerization, or monomer-dimer equilibrium and dimer addition mechanism, or conformation exchange), an elongation of the aggregates following a step, a linear or a power function, and a possible secondary process such as fragmentation.

1 Conversion of the Xue et al ODE model into a PDE model: example of the best-fit model.

Their best-fit model is given by the following processes:

- no conformational exchange, no coalescence and no degradation of polymers or monomers,
- the size of the nucleus is $i_0 = 2$ and denucleation occurs only through depolymerization,
- polymerization and depolymerization follow a step function with a step at $i = 6$,
- fragmentation into two smaller polymers occurs.

Thus, using the previously introduced notations, the original ODE system can be simplified as follows:

$$\frac{dc_1}{dt} = -i_0 k_{on}^N c_1^{i_0} + i_0 k_{off}^N c_{i_0} - c_1 \sum_{i \geq i_0} k_{on}^i c_i, \tag{1}$$

$$\frac{dc_{i_0}}{dt} = k_{on}^N c_1^{i_0} - k_{off}^N c_{i_0} - k_{on}^{i_0} c_1 c_{i_0} + 2 \sum_{j \geq i_0+2} k_{off}^{i_0,j} c_j \tag{2}$$

$$\frac{dc_i}{dt} = c_1 (k_{on}^{i-1} c_{i-1} - k_{on}^i c_i) - (k_{dep}^i c_i - k_{dep}^{i+1} c_{i+1}) + 2 \sum_{j \geq i+2} k_{off}^{i,j} c_j - K_{off}^i c_i. \tag{3}$$

Unlike the formulation of Xue et al., we did not introduce fragmentation into polymers of size 1, which can be included in the depolymerization terms. For the particular choice of fragmentation made in [1], however, fragmentation in polymers of size 1 is close to 0. This ODE system is then formally equivalent

to the following PDE system:

$$\frac{dc_1}{dt} = -\frac{i_0 k_{on}^N k_{on}(x_0) c_1^{i_0+1}}{k_{off}^N + k_{on}(x_0)c_1} - c_1 \int_{x_0}^{\infty} k_{on}(x)c(t,x)dx, \tag{4}$$

$$\frac{\partial c(t,x)}{\partial t} = -c_1 \frac{\partial}{\partial x}\big(k_{on}(x)c(t,x)\big) + \frac{\partial}{\partial x}\big(k_{dep}(x)c(t,x)\big) + 2\int_x^{\infty} k_{off}(x,y)c(t,y)dy - K_{off}(x)c(t,x), \tag{5}$$

$$c(t,x_0) = \frac{k_{on}^N c_1^{i_0}}{k_{off}^N + k_{on}(x_0)c_1}. \tag{6}$$

Note that our notations are slightly different from those presented in the SI of [1]. Due to the particular shape of the polymerization process, with a step at $i = 6$, it may be preferable to keep all the ODEs (3) occurring for $i \leq 6$ and consider the PDE (5) only for $i \geq 6$. Though nothing is indicated in [1], we suspect that the polymerization rate is much larger for $i \geq 6$ than for $i \leq 6$, and on the contrary that depolymerization is much smaller for larger i. If true, this would be interpreted as in some sense an energetic barrier or a kind of 'second nucleus' for $i = 6$. We then adapt the boundary condition (6) in the case where depolymerization and fragmentation for small polymers can be ignored and state, supposing $k_{on}^5 \ll k_{on}^6$ and thus an instantaneous equilibrium:

$$c(t,x_0) = \frac{k_{on}^5}{k_{on}^6} c_5, \tag{7}$$

c_5 given by Equation (3) taken for $i = 5$. A more detailed study would require knowing the order of magnitude of the best-fit parameter values.

2 Discussion on the Fragmentation Kernel

2.1 Discrete setting

The strategy developed in [1] to analyse the growth of amyloid fibrils consists in fitting transitional parameters of experimental reaction progress curves with 21 mathematical models combining pre-polymerization, elongation and fragmentation processes. In their papers, Xue and co-workers compare two different pre-polymerization and three different elongation functions, but only one fragmentation distribution according to the following equation based on statistical mechanical considerations for linear polymers [2]:

$$k_{off}^{j,i} = a\big(j(i-j)\big)^{b-1}\Big(\frac{(i-j)ln(j) + jln(i-j)}{i^{b+1}}\Big) \tag{8}$$

with $k_{off}^{i,j}$ the first order fragmentation rate of a species of size i into an aggregate of size j and an aggregate of size $j - i$, a the overall amplitude and b describing the size and position dependence of the fragmentation rate constant.

In order to generalize this approach to other fragmentation processes, we investigated the effect of the distribution of fragmentation on the transitional parameters, namely the length of the lag phase and the slope of the growth curve at the inflexion point. We simulated the following model (see main text for

notations and assumptions, equations [30]–[32]):

$$\frac{dc_1}{dt} = -i_0 k_{on}^N c_1^{i_0} + i_0 k_{off}^N c_{i_0} - c_1 \sum_{i \geq i_0} k_{on}^i c_i, \tag{9}$$

$$\frac{dc_{i_0}}{dt} = k_{on}^N c_1^{i_0} - k_{off}^N c_{i_0} - k_{on}^{i_0} c_1 c_{i_0} + 2 \sum_{j \geq i_0+2} k_{off}^{i_0,j} c_j \tag{10}$$

$$\frac{dc_i}{dt} = c_1(k_{on}^{i-1} c_{i-1} - k_{on}^i c_i) - (k_{dep}^i c_i - k_{dep}^{i+1} c_{i+1}) + 2 \sum_{j \geq i+2} k_{off}^{i,j} c_j - K_{off}^i c_i.$$

with two different distributions for the fragmentation : (i) the fragmentation rate defined by (8), as in [1], and (ii) a uniformly distributed fragmentation rate along the aggregates (*i.e.* $k_{off}^{j,i} = \frac{K_{off}^i}{i-1}$ constant for $j \leq i-1$). The total rate $K_{off}^i = \sum_j k_{off}^{j,i}$ with which a polymer of size i can fragmentate to give smaller aggregates was taken to be equal in both cases to study only the effect of the distribution of the fragmentation rate among smaller polymers:

$$K_{off}^i = \sum_{j \leq i} a\big(j(i-j)\big)^{b-1} \big(\frac{(i-j)ln(j) + jln(i-j)}{i^{b+1}}\big). \tag{11}$$

Figure S4 Left represents the typical shape of these two distributions for an aggregate of size $i = 20$. The other processes were taken as for the best fitting model of [1], *i.e.* no pre-polymerization and a fragmentation described by a step law. The numerical values used in the simulations were chosen to roughly reproduce the experimental curves of Xue et al [1]:

- $k_{on}^i = 0.00002\mu M^{-1}h^{-1}$ for $i < n_s$ and $0.9\mu M^{-1}h^{-1}$ for $i \geq n_s$
- $k_{on}^N = k_{on}^1$ and $k_{off}^N = k_{dep}^{i_0}$
- $k_{dep}^i = 0.5\mu M^{-1}h^{-1}$ for $i < n_s$ and $10^{-5}\mu M^{-1}h^{-1}$ for $i \geq n_s$
- $n_s = 6$
- $a = 0.0001\mu M^{-1}h^{-1}$
- $b = 5$
- $i_0 = 2$

Figure S4 Right represents the normalized reaction progress curves for three different initial concentrations ($50\mu M$, $100\mu M$ and $150\mu M$). Table S2 represents the transitional parameters of these curves, automatically extracted as described in [1]. One can observe graphically and numerically that these parameters are not very sensitive to the distribution of fragmentation.

2.2 Continuous setting

As stated above, the fragmentation kernel proposed by [1] is given by Equation (8). For a continuous model, a first attempt would be to simply replace $j < i$ by $x < y \in (0, +\infty)$. However, in such a case $ln(x)$ may become negative, so to avoid this we use the artefact of replacing $ln(j)$ by $ln(x+1)$, and $ln(i-j)$ by $ln(y-x+1)$. Indeed, in the original article [2] quoted by [1], polymers' diffusion coefficient are defined using a formula of Riseman and Kirkwood [3]. In this last article, ln appears through $ln(1/n)$, where n

being the number of monomers inside a polymer is supposed to be large, so that it is possible to replace it by $ln(1/(n+1))$. We thus obtain the following formula:

$$k_{off}(x,y) = a(x(y-x))^{b-1}\frac{(y-x)ln(1+x) + xln(1+y-x)}{y^{b+1}}. \quad (12)$$

In [1], fragmentation as a secondary process was requested to fit β_2-microglobulin experimental data. In addition, fragmentation seems to be a critical process in some others fibrillation processes, including prion strains [4]. Thus, developing a strategy that would help to characterize not only the magnitude of the fragmentation but also the distribution of this fragmentation along the aggregate size appears essential. Many studies suggest using the size distribution of aggregates to characterize more fully the distribution of elongation and fragmentation parameters [5–7] (see Figure S5), through PDE formalism and inverse problem techniques, for instance.

References

References

[1] Xue WF, Homans SW, Radford SE (2008) Systematic analysis of nucleation-dependent polymerization reveals new insights into the mechanism of amyloid self-assembly. PNAS 105: 8926–8931.

[2] Hill T (1983) Length dependence of rate constants for end-to-end association and dissociation of equilibrium linear aggregates. Biophysical Journal 44: 285 - 288.

[3] Riseman J, Kirkwood JG (1950) The intrinsic viscosity, translational and rotatory diffusion constants of rod-like macromolecules in solution. The Journal of Chemical Physics 18: 512-516.

[4] Zampieri M, Legname G, Altafini C (2009) Investigating the conformational stability of prion strains through a kinetic replication model. PLoS Comput Biol 5: e1000420.

[5] Knowles TPJ, Waudby CA, Devlin GL, Cohen SIA, Aguzzi A, et al. (2009) An Analytical Solution to the Kinetics of Breakable Filament Assembly. Science 326: 1533-1537.

[6] Calvez V, Lenuzza V, Oelz D, Deslys JP, Laurent P, et al. (2009) Size distribution dependence of prion aggregates infectivity. Math Biosci 1: 88–99.

[7] Calvez V, Lenuzza N, Doumic M, Deslys JP, Mouthon F, et al. (2010) Prion dynamic with size dependency - strain phenomena. J of Biol Dyn 4.

Figure Legends

Figure S4. Left: Size distribution of the fragmentation rate for an aggregation of size 20, following a uniform distribution (black) or a mechanical-based distribution (red) of fragmentation. **Right: Simulated normalized reaction progress curves of amyloid formation** for a uniform distribution (black) and a mechanical-based distribution (red) of fragmentation. See below for the numerical values.

Figure S5. Examples of simulated size distribution of the aggregates for a uniform distribution (black) and a mechanical-based distribution (red) of fragmentation. See above for the numerical values.

Tables

Table S2. Transitional parameters

Initial concentration	Parameter	Xue *et al.* fragmentation	Uniform fragmentation
$150\mu M$	T_{lag} (h)	0.189	0.189
	K $(\mu M.h^{-1})$	0.010	0.010
$100\mu M$	T_{lag} (h)	0.189	0.189
	K $(\mu M.h-1)$	0.007	0.007
$50\mu M$	T_{lag} (h)	13.91	14.23
	K $(\mu M.h^{-1})$	0.189	0.189

Transitional parameters extracted from the simulated reaction progress curves represented in Figure S4 Right
